# Dingzhen pills inhibit neuronal ferroptosis and neuroinflammation by inhibiting the cGAS-STING pathway for Parkinson’s disease mice

**DOI:** 10.1186/s13020-025-01135-9

**Published:** 2025-06-16

**Authors:** Gaoshuang Fu, Ting Li, Yukun Zhao, Shuai Zhang, Xin Xue, Yuqin Yang, Tingyu Li, Shaohan Luo, Guangxin Yue, Tong Lei

**Affiliations:** 1 Institute of Basic Theory for Chinese Medicine, China Academy of Chinese Medical Sciences, Beijing, 100700 China; 2 Dongzhimen Hospital, Beijing University of Chinese Medicine, Beijing, 100700 China

**Keywords:** Parkinson’s disease, Proteomics, Traditional Chinese Medicine, Neuroinflammation, Ferroptosis, Network pharmacology

## Abstract

**Background:**

Parkinson’s disease (PD) is a neurodegenerative disease whose cause and molecular mechanism remain unclear. Dingzhen pill (DZP), a water extract from crude herbs, is thought to possibly play a neuroprotective role in PD. However, the underlying pharmacological mechanisms of the DZP and its impact on the potential pathways against PD have not been elucidated.

**Aim:**

The aim of this study was to explore the effect and molecular mechanism of DZP treatment on PD by quantitative proteomic and metabolomic analysis.

**Methods:**

The effect of DZP on the behavior of movement disorders in a mouse model of MPTP-induced was first evaluated. Secondary metabolomics for TCM resolved the components of DZP and its metabolites distributed in the serum of PD mice. Proteomics was used to resolve the effects of DZP on proteins in brain tissue in mice with PD. Network pharmacology and molecular docking analysis were used to screen and validate the binding relationships of components and targets. Immunofluorescence and western blot analysis verified the pathway affected by DZP.

**Results:**

Our study showed that DZP improved the movement disorder of PD mice and promoted the recovery of dopaminergic neurons in substantia nigra. Secondary metabolomics showed that 460 kinds of secondary metabolites existed in serum of PD mice after DZP treatment. Proteomics analysis showed that DZP affected the expression of 9393 proteins, 87 of which were up-regulated and 120 down-regulated. Through target prediction and molecular docking, a total of 8 compounds were found to interact with 8 pathological PD targets, playing a significant role in neuronal protection. In addition, DZP inhibits neuronal ferroptosis and neuroinflammation by inhibiting the cGAS-STING pathway.

**Conclusion:**

This study is helpful to understand the molecular mechanism of the multi-component, multi-target neuroprotective effect of DZ, and may contribute to the development of DZP as a new therapeutic strategy for clinical application in PD.

**Supplementary Information:**

The online version contains supplementary material available at 10.1186/s13020-025-01135-9.

## Introduction

Parkinson’s disease (PD) is the second most prevalent neurodegenerative disorder globally and the fastest growing neurologic disease [1]. Current estimates indicate over 6 million cases worldwide, projected to double by 2040 [2]. Clinical manifestations of PD include motor symptoms such as tremor, rigidity, bradykinesia, and postural instability, as well as non-motor symptoms like sleep disturbances, olfactory dysfunction, autonomic dysfunction, cognitive impairment, and psychiatric disorders [3]. Pathological changes in PD involve degeneration of dopaminergic neurons in the substantia nigra and the formation of Lewy bodies in residual neurons. Biochemical alterations include dopamine depletion in the striatum and imbalance between dopamine and acetylcholine neurotransmission [4]. The pathogenesis of PD is associated with various mechanisms including abnormal aggregation of α-synuclein (α-syn), mitochondrial dysfunction, oxidative stress, immune activation, neuroinflammation, lysosomal dysfunction, impaired endocytosis and cellular transport, and gut microbiota dysbiosis [4]. Current pharmacological treatments for PD include levodopa (L-Dopa), dopamine receptor agonists, monoamine oxidase type B inhibitors, catechol-O-methyltransferase inhibitors, anticholinergics, and amantadine [5]. However, these medications may lead to adverse effects such as dyskinesia, psychiatric symptoms, and gastrointestinal reactions, which exacerbate patient distress and reduce medication compliance [5].

In recent years, given the high complexity of neurodegenerative diseases, multi-target drug development has been proposed [6]. Consequently, multi-component and multi-target of Traditional Chinese Medicine (TCM) has garnered significant attention for their neuroprotective effects in PD [7, 8]. Dingzhen pill (DZP) is a renowned TCM formula composed of Astragalus, Atractylodes, White Peony Root, Rehmannia (both cooked and raw), Angelica sinensis, Chuanxiong, Gastrodia Elata, Gentiana macrophylla, Clematis, Saposhnikovia, Schizonepeta, Asarum, and Scorpion. Several clinical studies have demonstrated the efficacy of DZP in treating PD [9, 10]. And the combination of DZP and pramipexole for treating PD patients resulted in significantly lower UPDRS scores compared to the use of pramipexole alone [11]. In PD rat model, DZP was found to modulate neurotransmitters and reduce oxidative stress in dopaminergic neurons of the substantia nigra [12]. However, the neuroprotective effects of DZP on PD are still not well understood. Network pharmacology and multi-omics technologies have made it possible to quickly and accurately identify the targets of TCM [13]. This study aim to explore the potential active ingredients of DZP and its neuroprotective pharmacological mechanism in the treatment of PD through secondary metabolomics, proteomics and network pharmacology.

## Materials and methods

### Reagents

Daidzein, Albiflorin, Pinobanksin, 3-(3-Hydroxyphenyl) propanoic acid, Ursolic acid, Paeoniflorin, Mangiferolic acid and Morolic acid purchased from Macklin Company (Shanghai, China). MPTP and 1-Methyl-4-phenylpyridinium iodide (MPP +) were obtained from Sigma-Aldrich (St. Louis, MO, USA). The cell counting kit (CCK8), 4',6-diamidino-2-phenylindole (DAPI) obtained from Beyotime Biotechnology (Shanghai, China). Antibodies including TH, α-syn, GFAP, TNF-α, NeuN, GPX4 and conjugated secondary antibodies were bought from Servicebio (Wuhan, China).

### Mice and MPTP-induced PD mouse model

**Fig. 1:**
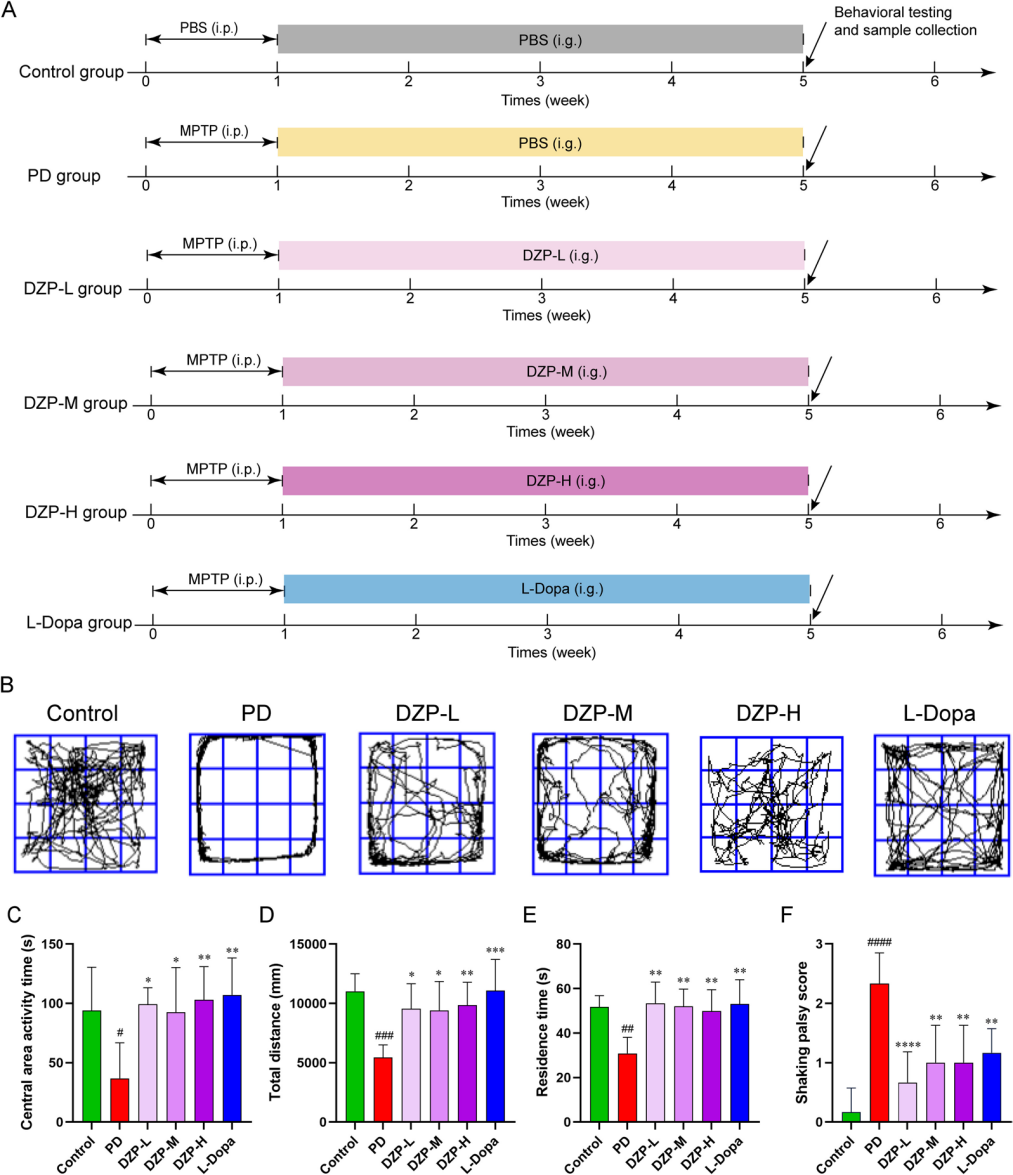
DZP improves mobility disorders in a mouse model of PD. **A** Grouping and treatment protocol for DZP used to treat PD mice. **B** Trajectory map of mouse movement in an open field. **C** Time spent in the central area of the open field by mice. **D** Total distance traveled by mice in the open field. **E** Time spent by mice on the rotarod test. **F** All mice were scored in the Shaking palsy score test. n = 10. The data are mean ± SD; #*P* < 0.05; ##*P* < 0.01; ###*P* < 0.001 compared to the PD group.*P < 0.05; **P < 0.01; ***P < 0.001 compared to the PD group

All animal procedures were evaluated and approved by the Experimental Animal Welfare and Ethics Committee of the Institute of Basic Theory for Chinese Medicine, China Academy of Chinese Medical Sciences. Male C57BL/6 mice (6–8 week, 20 ± 2 g) were purchased from Charles River (Beijing, China) and were housed in SPF-grade animal rooms with a temperature of 22 ± 2 °C, humidity of 55 ± 5%, with a 12 ± 2 h light/dark cycle. Free access to food and water. All mice were randomly divided into 6 groups (n = 10). Mice of PD group received intraperitoneal injections of MPTP (25 mg/kg/day), 7 days; Mice of DZP-Low (DZP-L, 6.25 g/kg/day), medium (DZP-M, 12.5 g/kg/day), high (DZP-H, 25 g/kg/day) group received gavage DZP after injections of MPTP; and mice of the L-Dopa group received gavage (20 mg/kg/day) of L-Dopa (Fig. 1A).

### Open field test

The open field test apparatus has a closed bottom and sides with an open top and is white inside. The internal dimensions are 40 cm (length) × 40 cm (width) × 40 cm (height). The central area of the floor, measuring 20 cm × 20 cm, is designated as the central zone, while the surrounding area is the peripheral zone. A camera mounted directly above the open field is connected to a computer-based tracking analysis system. During the experiment, each mouse is placed in the center of the open field and allowed to move freely for 5 min. The system records the mouse’s movement speed, distance traveled, movement trajectory, and time spent in the central zone. Between tests, the open field apparatus is wiped with 75% alcohol to remove any residual odors.

### Rotating rod test

First, mice are trained to maintain their balance on a rotating rod and to stay on the rod rotating at 4 rpm for at least 60 s. Once this level of proficiency is achieved, the testing begins. During the test, the acceleration parameter is set to gradually increase the rotation speed from 4 to 40 rpm over 5 min, until the mouse falls off the rod. The time each mouse stays on the rod before falling is recorded. Each mouse is tested three times with intervals of more than 1 h between tests. The recorded data is then analyzed.

### Shaking palsy test

Place the mice in the behavioral observation box and allow them to acclimate to the environment for 5–10 min to reduce the influence of environmental factors on the test results. During this period, observe the general behavior of the mice to ensure that they have no abnormal behavior. The tester observes the tremors of the mice with the naked eye and scores them using specific scoring criteria. Usually, the scoring criteria are as follows: 0 points: no tremors; 1 point: slight tremors occur occasionally and do not affect the normal activities of the mice; 2 points: slight tremors occur frequently, but the mice can still move normally; 3 points: moderate tremors have a certain impact on the activities of the mice, such as slight shaking of the body when walking; 4 points: severe tremors seriously affect the activities of the mice, and the mice have difficulty walking or standing normally. Observe and record the tremors of the mice within 3–5 min, and take the average score as the tremor score of the mouse. During the test, keep the environment quiet and avoid external interference.

### Secondary metabolomics analysis of TCM

Add all samples to 300 μL of 70% methanol extraction solution containing internal standards. Centrifuge at 12,000 rpm for 10 min at 4 °C. Samples were analyzed using Ultra Performance Liquid Chromatography (UPLC) coupled with Tandem Mass Spectrometry (MS/MS). The chromatography column used was an Agilent SB-C18, 1.8 μm, 2.1 mm × 100 mm. The mobile phase consisted of: A) ultrapure water with 0.1% formic acid; and B) acetonitrile with 0.1% formic acid. The liquid phase gradient was as follows: 0–9.0 min, linear gradient of phase B from 5 to 95%; 9.1–10 min, phase B maintained at 95%; 10.1–11.1 min, linear gradient of phase B from 95 to 5%; 11.2–14 min, phase B maintained at 5%. The flow rate was 0.35 mL/min; column temperature was 40 °C; and the injection volume was 2 μL. The analysis used the multiple reaction monitoring (MRM) mode of a triple quadrupole mass spectrometer with medium collision nitrogen gas. Specific MRM ion pairs were monitored based on the eluted metabolites during each period. Differential metabolites were screened using Orthogonal Partial Least Squares-Discriminant Analysis (OPLS-DA) to analyze the metabolomics data. Initially, Variable Importance in Projection (VIP) scores obtained from the OPLS-DA model were used to identify potential differential metabolites between the PD group and the DZP group. This was further refined by incorporating Fold Change (FC) values. Metabolites with VIP > 1 and FC ≥ 2 or FC ≤ 0.5 were selected as differential metabolites. Additionally, these metabolites were required to be present in the DZP herbal decoction samples.

### Target prediction of TCM

The differential metabolites identified were imported into PubChem for information retrieval. Potential target molecules of the active compounds were predicted using the following databases: Batman-TCM (http://bionet.ncpsb.org.cn/batman-tcm/), PharmMapper (https://www.lilab-ecust.cn/pharmmapper/), TargetNet (http://targetnet.scbdd.com/), and SwissTargetPrediction (http://www.swisstargetprediction.ch/).

### Proteomics

Midbrain tissues from PD group and DZP group mice were collected, and an appropriate amount of SDT lysis buffer was added. The samples were then boiled for 3 min and ultrasonically disrupted for 2 min. The samples were centrifuged at 16,000 g for 20 min at 4 °C, and the supernatant was collected. Protein was subjected to Filter-aided Sample Preparation for digestion. The resulting peptides were desalted using a C18 cartridge and lyophilized. The dried peptides were reconstituted in 0.1% formic acid and their concentration was measured in preparation for LC–MS analysis. Chromatographic separation was performed using a Vanquish Neo UHPLC system (Thermo Scientific). The mobile phase consisted of: A) ultrapure water with 0.1% formic acid; and B) acetonitrile with 0.1% formic acid. After peptide separation, data independent acquisition (DIA) was performed using an Orbitrap Astral mass spectrometer (Thermo Scientific). All mass spectrometry data were merged and analyzed using the DIA-NN 1.8.1 software. Database search and protein DIA quantification were conducted with a Q-value threshold of ≤ 0.01. The database used was uniprot-Mus musculus (Mouse). The experimental data were filtered based on general principles. Proteins were selected if they had at least 50% non-missing values in the corresponding sample groups, with missing values in the remaining data filled in.

### Bioinformatics analysis

PD disease targets were searched using the OpenTargets (https://www.opentargets.org/), CTD (http://ctdbase.org/), GeneCards (http://genecards.org/), and DisGeNET (https://www.disgenet.org/) databases. Gene names were aggregated and standardized to obtain disease targets. Using the R 1.7.3 software with the VennDiagram package, a Venn diagram was constructed to visualize the overlap between the predicted targets from metabolite analysis and PD disease targets, resulting in a list of intersecting targets. Subsequently, the ‘Component-Target-Disease’ network was visualized using Cytoscape 3.9.1. Protein–protein interaction (PPI) networks were constructed using the STRING database and Cytoscape to identify core targets within the intersecting target list. Gene Ontology (GO) enrichment analysis and Kyoto Encyclopedia of Genes and Genomes (KEGG) pathway enrichment analysis were performed using the R 4.6.2 software with the clusterProfiler package to explore the potential mechanisms of action of the compounds. Additionally, potential targets of DZP treatment for PD were also identified from proteomics results. GO enrichment analysis and KEGG pathway enrichment analysis were conducted using the same methods to explore these targets.

### Molecular docking

Important compounds were docked with core target molecules. The 3D structures of the main active compounds were downloaded from the PubChem database, and the 3D structures of the core target proteins were obtained from the Protein Data Bank (https://www.rcsb.org/). The core target proteins were initially processed to remove solvent molecules and other components using PyMOL 2.6.0. AutoDock Tools 1.5.7 was then used for further preparation, including adding hydrogens and charges. The core target proteins and active compounds were saved in ‘pdbqt’ format, with appropriate grid positions and sizes set. Docking of the compounds and targets was performed using AutoDock Vina, and the docking results were visualized using PyMOL.

### Content detection of Iron, MDA and ROS

To measure the iron content in midbrain tissues using the Iron Content Assay Kit (Solarbio, China), start by thoroughly lysing the cells. Following cell lysis, centrifuge the samples to remove any debris, collecting the clear supernatant for analysis. Add the provided detection reagent to the supernatant according to the kit’s instructions, ensuring precise pipetting for accurate results. Incubate the mixture for a specifed duration under the recommended conditions to allow for the complete reaction between sample and the detection reagent. After incubation, measure the absorbance of the samples using a spectrophotometer (CMaxPlus, MD, Shanghai) at the wavelength of in the kit’s protocol. Absorbance was measured at 532 nm to detect iron levels, 495/530 nm excitation/emission wavelengths to detect MDA levels; and 488/525 nm excitation/emission wavelengths to detect ROS levels, respectively.

### Cell culture and viability

Human neuroblastoma cells SH-SY5Y was seeded in DMEM medium supplemented with 10% fetal bovine serum (FBS, BI, Israel), 1% 10,000 units/mL penicillin and 1% 10 mg/mL streptomycin (BI, Israel), and 100 μmol L-ascorbic acid (BI, Israel). Place the flask (Corning, USA) in a constant temperature incubator at 37 °C with 100% humidity and 5% CO_2_ concentration. Replace with fresh medium every 2–3 days and passage after a cell confluency of 80–90%. The in vitro models of PD constructed by pretreating SH-SY5Y cells with MPP + (4 mM) for 24 h, the effect of nanoparticles on cell viability was detected by CCK8 colorimetry. A total of 2 × 10^5^ cells/mL of SH-SY5Y cells was seeded into 96-well plates, incubated at 37 °C for 1 h with CCK8 solution after 24 h of different drug treatment, and finally measured by microplate reader the absorbance generated at 450 nm. A total of 3 independent experimental replicates were performed.

### Immunofluorescence

Mice brain tissues were fixed in 4% paraformaldehyde for 48 h and then stored in 30% sucrose solution at 4 °C. Coronal sections with a thickness of 20 μm were cut, and sections from the substantia nigra were collected. The sections were then incubated sequentially with primary antibodies against Tyrosine Hydroxylase (TH) (Proteintech, Wuhan, China), GFAP(Proteintech, Wuhan, China), TNF-α (Proteintech, Wuhan, China), NeuN (Proteintech, Wuhan, China), GPX4 (Proteintech, Wuhan, China) and α-syn (Proteintech, Wuhan, China), followed by incubation with fluorescence-labeled secondary antibodies (Proteintech, Wuhan, China). The high-resolution immunofluores cence results were scanned and saved using a confocal microscope (Leica Biosystems). The intensity of the fluorescence was detected in the chemiluminescence analyzer and analyzed with ImageJ software.

### Western blot

The mouse midbrain tissues were homogenized in lysis buffer (RIPA: PMSF: protease inhibitor = 100:1:1) to extract total protein. Protein concentration was measured using a BCA assay kit. Equal amounts of protein samples were separated by 10%/15% SDS-PAGE and transferred to a PVDF membrane (Millipore). The membrane was blocked with 5% skim milk at room temperature for 1 h, followed by overnight incubation at 4 °C with primary antibodies against ACSL4 (ABclonal, Wuhan, China), TH (Proteintech, Wuhan, China), SLC7 A11 (ABclonal, Wuhan, China), GPX4 (ABclonal, Wuhan, China), and β-actin (ABclonal, Wuhan, China). Afterward, the membrane was incubated with horseradish peroxidase-conjugated secondary antibodies (ABclonal, Wuhan, China) at room temperature for 1 h. Antigen–antibody complexes were detected using enhanced chemiluminescence (ABclonal, Wuhan, China) and visualized with the ChemiDoc MP system (Bio-Rad, USA). Results were quantified using ImageJ software. The experiment was conducted in triplicate.

### Statistical analysis

Statistical analyses and graphing were performed using GraphPad Prism 9 (GraphPad, San Diego, California, USA). Quantitative data are expressed as mean ± standard deviation. The t-test was used for comparisons between the two groups, and one-way ANOVA was used for three or more groups. A P-value of < 0.05 was considered statistically significant, with * *P* < 0.05, ** *P* < 0.01, and *** *P* < 0.001 indicating levels of significance.

## Results

### DZP can improve the movement disorder of PD mice

We first examined the pharmacodynamic effects of DZP in PD mice. We first evaluated the efficacy of DZP in treating MPTP-induced PD mice. After a week of MPTP intraperitoneal injection to create a PD mouse model, DZP was administered orally for 4 weeks. After treatment, the movement impairment of PD mice was evaluated by open field test, rotarod test and shaking palsy test. The results showed that the speed, total distance, and time spent in the central area of PD mice were significantly reduced compared to the control group, while they were significantly increased after DZP treatment (Fig. 1B–E). In addition, DZP treatment significantly increased the time spent by PD mice on the rotarod (Fig. 1F). Meanwhile, the shaking palsy score in PD mice significantly increased, and this score decreased after DZP treatment (Fig. 1G).These research results indicate that DZP treatment significantly improves the mobility disorders of PD mice.

### DZP repaired TH neurons in the substantia nigra region of PD mice

**Fig. 2:**
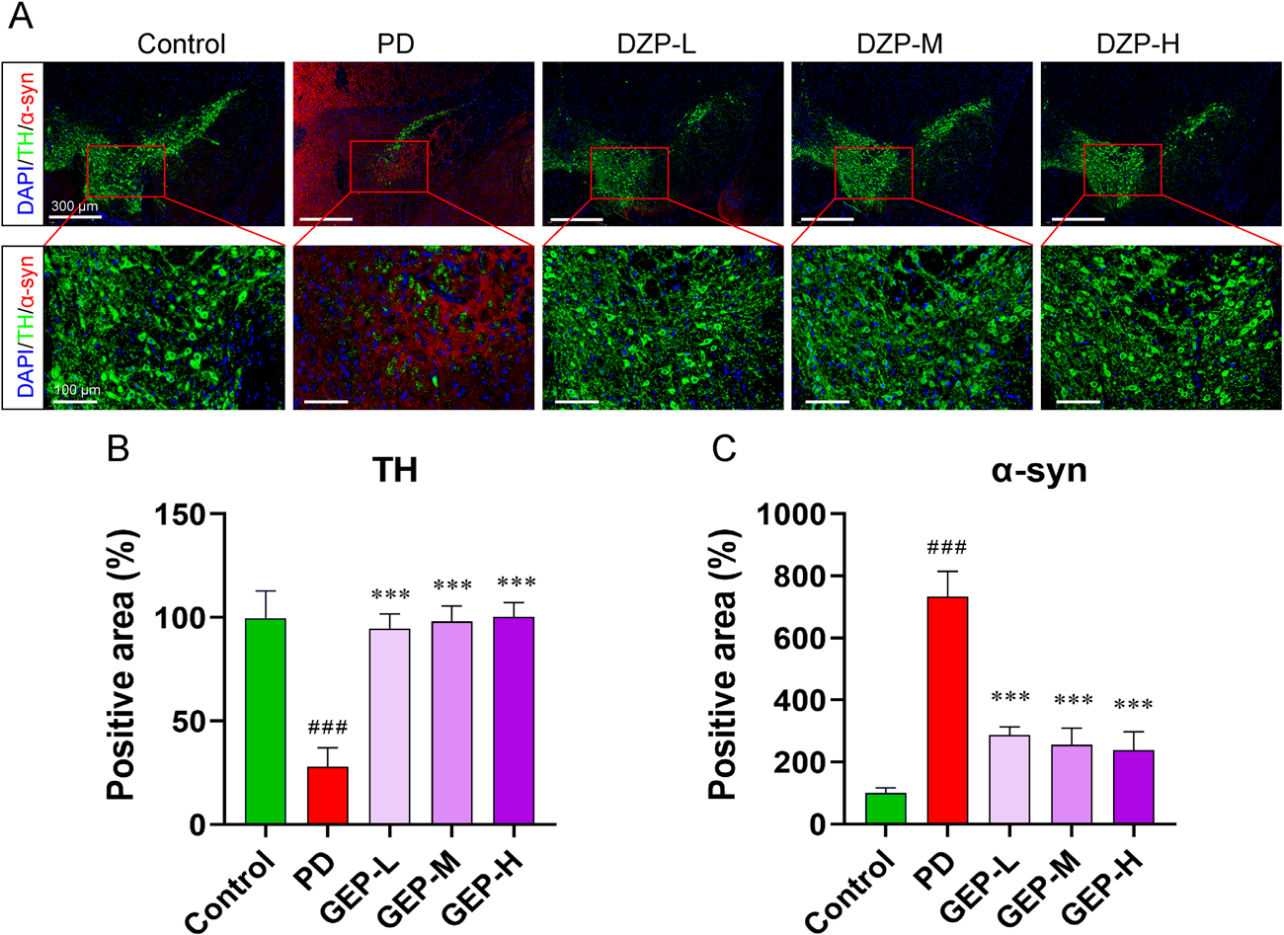
Effect of DZP treatment on midbrain tissue in PD mice. **A** Immunofluorescence results of TH and α-syn in the substantia nigra of mouse midbrain. Scale bars: 300 μm or 100 μm. **B** Statistical map of TH positive expression area. **C** Statistical map of α-syn positive expression area. n = 10. The data are mean ± SD; #*P* < 0.05; ##*P* < 0.01; ###*P* < 0.001 compared to the PD group.*P < 0.05; **P < 0.01; ***P < 0.001 compared to the PD group

The pathological features of PD patients include damage to TH neurons in the substantia nigra and accumulation of α-syn. Immunofluorescence analysis was used to detect the expression of TH and α-syn in the substantia nigra region. The results showed that compared to the healthy group, the expression of TH in the substantia nigra region of PD mice was significantly decreased, while the expression of α-syn was significantly upregulated. After treatment with DZP, the damage to TH neurons in PD mice was restored, and α-syn was significantly downregulated (Fig. 2). These results indicate that DZP treatment improved TH neurons in the substantia nigra region of PD mice.

### Compound analysis of DZP for PD mice

**Fig. 3:**
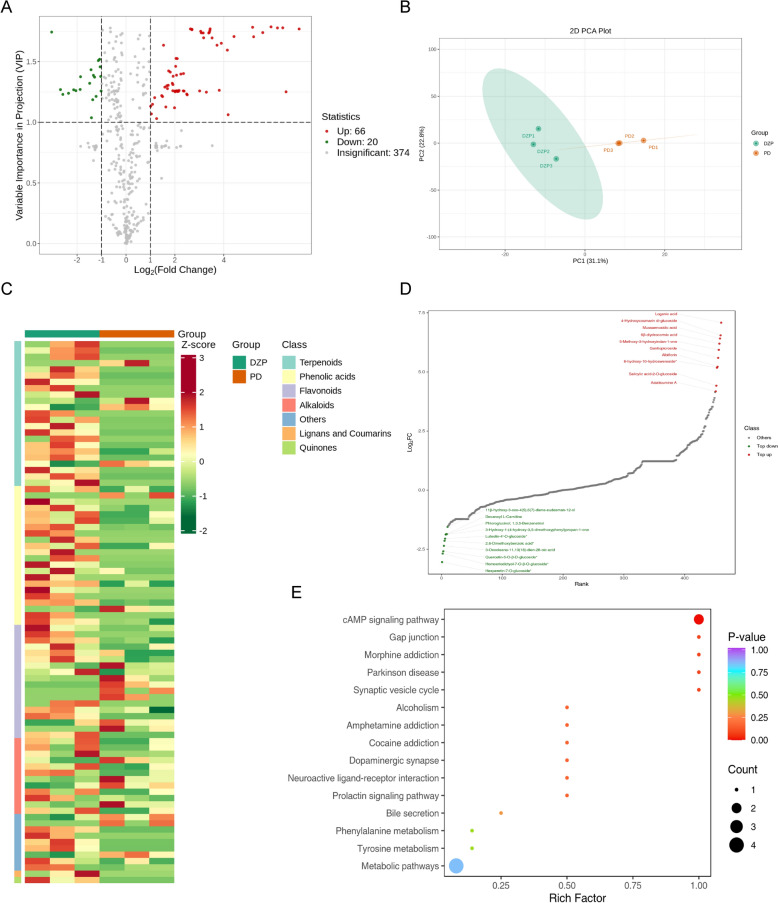
Identification of DZP components and its components in serum of PD mice. **A** Differentially expressed metabolites volcano plot. **B** Principal component analysis of differentially expressed metabolites. **C** Heat map of differentially expressed metabolites. **D** Ranking of differentially expressed metabolites. **E** KEGG pathway analysis of differentially expressed metabolites

To elucidate the potential mechanisms by which DZP exerts its therapeutic effects in vivo, we detected secondary metabolites in the mouse serum. Results showed that a total of 460 secondary metabolites were detected (Fig. S1), of which 66 were upregulated and 20 were downregulated (Fig. 3A, Table 1). The Principal Component Analysis results showed that differential metabolites could significantly distinguish the treatment group from the PD model group (Fig. 3B). A heat map was drawn to show that DZP treatment group upregulated several small molecules, including Terpenoids, Phenolic acids, and Flavonoids (Fig. 3C). KEGG analysis revealed that these metabolites are involved in several important pathways, including Phenylalanine metabolism, Metabolic pathways, Tyrosine metabolism, Neuroactive ligand-receptor interaction, Synaptic vesicle cycle, Dopaminergic synapse, and Parkinson’s disease (Fig. 3E).

**Table 1:** Serum composition analysis DZP was used after feeding PD mice

Compounds	Formula	CAS
1,3,6-trihydroxy-7-methoxy-2-{[(2 s,3r,4 s,5 s,6r)−3,4,5-trihydroxy-6-(hydroxymethyl)oxan-2-yl]oxy}xanthen-9-one	C_20_H_20_O_12_	–
1-Methyl-6-Oxo-1,6-Dihydropyridine-3-Carboxamide	C_7_H_8 N2_O_2_	701–44-0
1-O-p-Coumaroyl-β-d-glucose	C_15_H_18_O_8_	7139–64-2
24,30-Dihydroxy-12(13)-ene-lupeol	C_30_H_48_O_3_	–
2-Phenylethyl beta-primeveroside	C_19_H_28_O_10_	129,932–48-5
3-(3-Hydroxyphenyl)-propionic acid	C_9_H_10_O_3_	621–54-5
3,5,7-Trihydroxyflavanone (pinobanksin)	C_15_H_12_O_5_	548–82-3
3-Aminosalicylic acid	C_7_H_7 N_O_3_	570–23-0
3-Galloylquinic acid	C_14_H_16_O_10_	17,365–11-6
3-Hydroxy-9,19-cyclolanost-24-en-26-oic acid (Mangiferolic acid)	C_30_H_48_O_3_	4184–34-3
3-Hydroxyphenylacetic acid methyl ester	C_9_H_10_O_3_	42,058–59-3
3-Hydroxyurs-12-en-28-oic acid (Ursolic acid)	C_30_H_48_O_3_	77–52-1
3-O-Methylgallic acid	C_8_H_8_O_5_	3934–84-7
3-O-p-Coumaroylquinic acid	C_16_H_18_O_8_	87,099–71-6
4-Hydroxycoumarin di-glucoside	C_21_H_26_O_13_	–
4-O-Galloylquinic acid	C_14_H_16_O_10_	110,170–37-1
5'-Glucosyloxyjasmanic acid	C_18_H_28_O_9_	–
5-Methoxy-3-hydroxyindan-1-one	C_10_H_10_O_3_	–
5-O-p-Coumaroylquinic acid	C_16_H_18_O_8_	1899–30-5
6a-dihydrocornic acid	C_16_H_24_O_10_	–
6-hydroxy-2-(3-methoxybenzylidene)−1-benzofuran-3(2H)-one	C_16_H_12_O_4_	1,234,351–88-2
6-Hydroxy-2'-methoxyflavone	C_16_H_12_O_4_	61,546–59-6
6'-O-BenzoyI-8-epiloganic acid	C_23_H_28_O_11_	–
6-O-Caffeoylarbutin	C_21_H_22_O_10_	136,172–60-6
6β-diydrocormic acid	C_16_H_24_O_10_	–
8-hydroxy-10-hydrosweroside	C_16_H_24_O_10_	–
9-[(2S,3S,4S,5R)−3,4-dihydroxy-5-(hydroxymethyl)oxolan-2-yl]−1H-purin-6-one	C_10_H_12 N4_O_5_	–
Acetylcatalpol	C_17_H_26_O_12_	–
Ajugol	C_15_H_24_O_9_	52,949–83-4
Albiflorin	C_23_H_28_O_11_	39,011–90-0
Asiaticumine A	C_16_H_13 N_O_4_	–
Biochanin A-7-O-glucoside-6''-O-malonate	C_25_H_24_O_13_	34,232–17-2
Calycosin-7-O-glucoside	C_22_H_22_O_10_	20,633–67-4
Daidzein	C_15_H_10_O_4_	486–66-8
demethylsecologanol	C_16_H_24_O_10_	–
Dihydroferulic acid	C_10_H_12_O_4_	1135–23-5
Dihydroferulic acid glucoside	C_16_H_22_O_9_	–
Dihydrojuglone	C_10_H_8_O_3_	–
Dopamine	C_8_H_11 N_O_2_	51–61-6
Ferulic acid methyl ester	C_11_H_12_O_4_	2309–07-1
Ferulic β-glucoside	C_16_H_20_O_9_	14,364–12-6
Feruloyl syringic acid	C_19_H_18_O_8_	–
Gardoside	C_16_H_22_O_10_	54,835–76-6
Gentiopicroside	C_16_H_20_O_9_	20,831–76-9
Glucosyl 3,7-dimethylocta-2,6-diene-1,4,8-trio	C_16_H_28_O_8_	–
glycoric acid	C_12_H_20_O_4_	–
Hydroxymenisdaurin D	C_14_H_21 N_O_8_	–
Loganic acid	C_16_H_24_O_10_	22,255–40-9
Monomelittoside	C_15_H_22_O_10_	20,633–72-1
Morolic acid	C_30_H_48_O_3_	559–68-2
Mussaenosidic acid	C_16_H_24_O_10_	82,451–22-7
Naringenin-4'-O-glucoside	C_21_H_22_O_10_	–
Oxyallobetulin	C_30_H_48_O_3_	–
Paeoniflorin	C_23_H_28_O_11_	23,180–57-6
paeonisothujone	C_10_H_14_O_3_	158,204–37-6
p-Coumaric acid methyl ester	C_10_H_10_O_3_	19,367–38-5
p-Coumaric acid-4-O-glucoside	C_15_H_18_O_8_	117,405–48-8
Phenylethanolamine	C_8_H_11 N_O	7568–93-6
Poncirin (Isosakuranetin-7-O-neohesperidoside)	C_28_H_34_O_14_	14,941–08-3
Prunetin-4'-O-glucoside(Prunitrin)	C_22_H_22_O_10_	154–36-9
Pseudotropine	C_8_H_15 N_O	135–97-7
Salicylic acid-2-O-glucoside	C_13_H_16_O_8_	10,366–91-3
Scutellarein-7-O-glucuronide (scutellarin)	C_21_H_18_O_12_	27,740–01–8
Swertiamarin	C_16_H_22_O_10_	17,388–39-5
Syringaldehyde; 4-Hydroxy-3,5-Dimethoxybenzaldehyde	C_9_H_10_O_4_	134–96-3
Vanillylamine	C_8_H_11 N_O_2_	1196–92-5

### Target with PD analysis of components

**Fig. 4:**
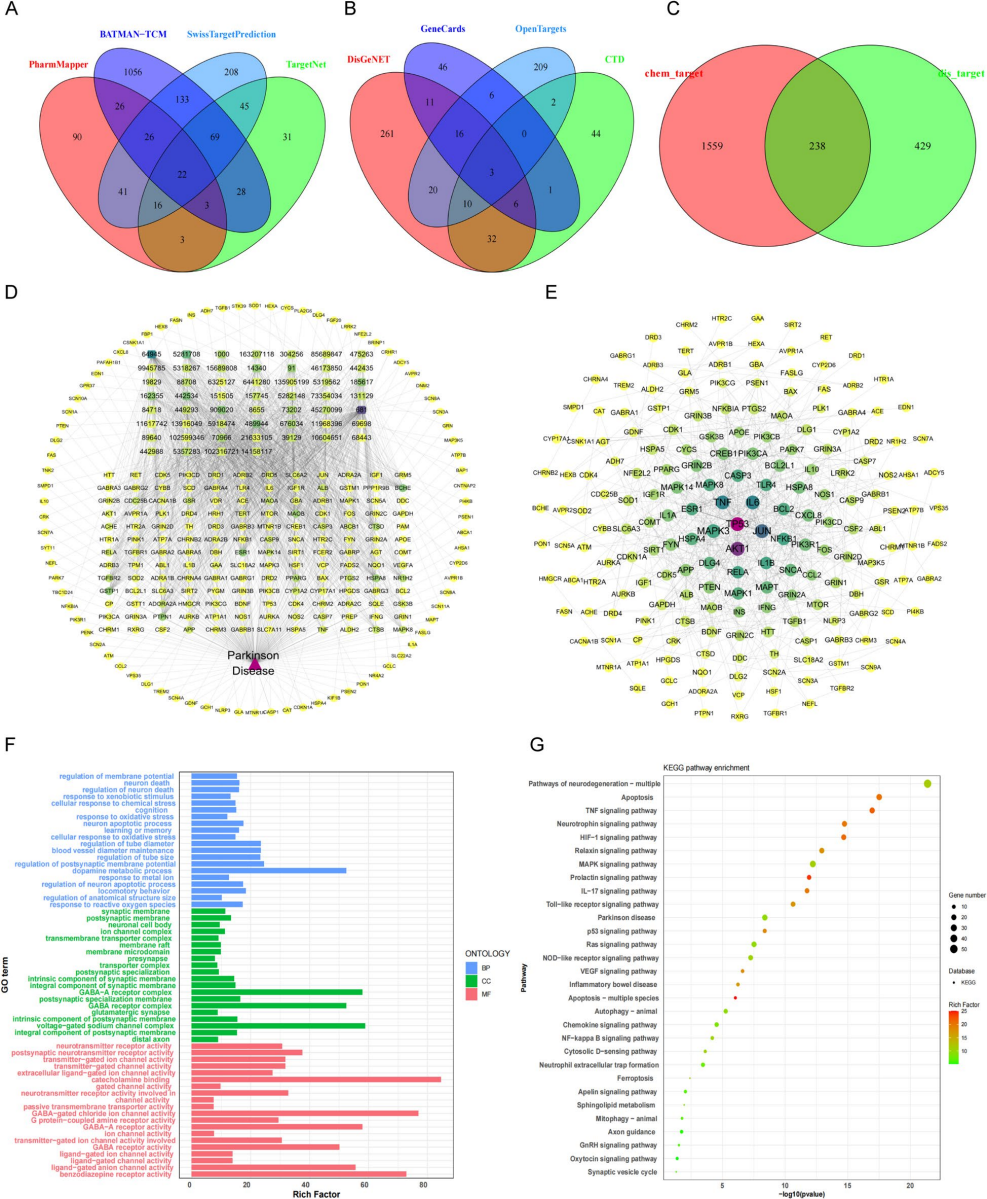
Potential targets analysis of the compounds of DZP. **A** Targets of compounds from different databases. **B** PD disease targets from different databases. **C** Intersection of compound targets and PD disease targets. **D** Interaction network of compound targets and PD disease targets. **E** Interaction network of compound targets with PD pharmacological targets. **F** GO analysis of compound targets. **G** KEGG analysis of compound targets

To understand the potential targets and pathways of these small molecules, we used PubChem to search for effective information on the compounds. We predicted the potential target molecules of the active components of the compounds using Batman-TCM (Probability ≥ 0.6), PharmMapper (Probability ≥ 70%), TargetNet (Probability ≥ 0.7), SwissTargetPrediction (Probability ≥ 0), and obtained 1797 potential compound targets (Fig. 4A). We then searched for PD disease results using OpenTargets (Score ≥ 0.4), CTD (Score ≥ 80), GeneCards (Score ≥ 50), and DisGeNET (Score ≥ 0.1); we screened the disease targets according to the threshold and summarized and standardized the gene names to obtain 667 disease targets (Fig. 4B). We selected the potential PD targets and obtained 238 common action targets by intersecting the 66 compounds in DZP with the 1797 potential targets of the compounds, thus identifying the potential targets of the compounds acting on the disease (Fig. 4C). The compound-target-disease network was mapped, with 292 nodes, 1537 edges, where the edges represent the targeting relationships between compounds and targets (Fig. 4D). Among the 53 compounds, 238 targets were targeted, to some extent reflecting the mechanisms of TCM through different active ingredients acting on the same target, and the same active ingredient acting on multiple targets. The network topology properties were analyzed using the Network Analyzer plug-in, where the size of the nodes represents the degree value of the node, and the degree value is larger, indicating that the node has a stronger hub role in the network. Based on this analysis, the main active ingredients were identified as dopamine, Ursolic Acid, Daidzein, Paeoniflorin, (2Z)−6-hydroxy-2-(3-methoxybenzylidene)−1-benzofuran-3(2H)-one, etc. Subsequently, the intersection of 238 compounds with disease-related targets was imported into the STRING database to obtain PPI relationships and STRING interaction graph, where there were 194 nodes and 593 edges. The core targets were found to be TP53, AKT1, JUN, IL6, TNF, MAPK3, NFKB1, etc., which were the key targets for the 66 compounds in DZP for disease treatment (Fig. 4E). Based on R clusterProfiler, the 238 compounds with disease-related intersection targets were subjected to GO enrichment analysis, and the top 15 entries in the biological process (BP), molecular function (MF), and cellular component (CC) were selected and plotted as bar charts (Fig. 4F). We conducted KEGG pathway enrichment analysis on the target points, resulting in the enrichment of 196 signaling pathways, mainly involving ferroptosis, Parkinson’s disease, etc. (Fig. 4G). The pathways work together to treat diseases through synergistic effects.

### DZP regulates the expression of protein in PD mice

**Fig. 5:**
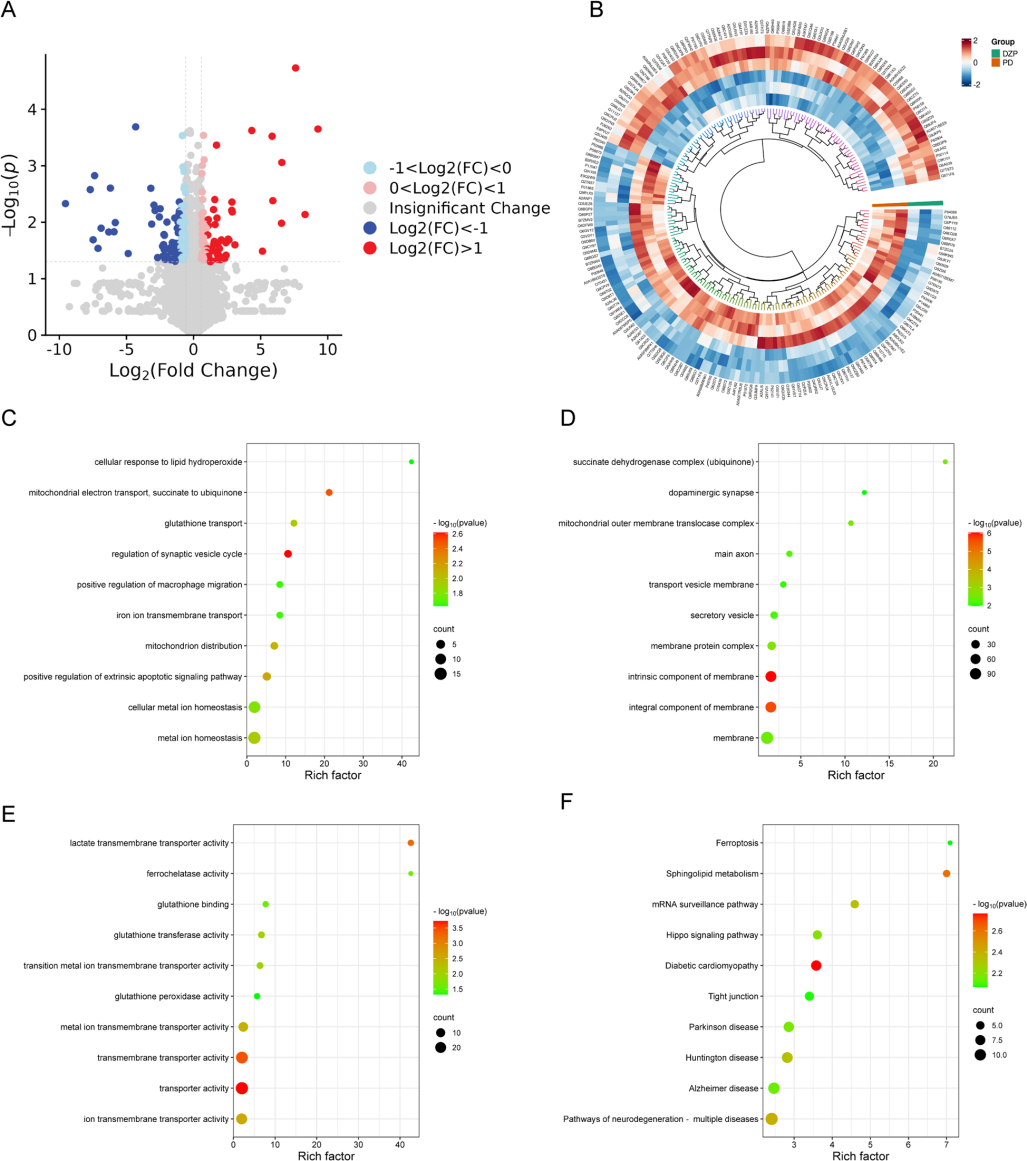
Proteinomics analysis of the midbrain tissue in PD mice treated with DZP. **A** Volcano plot of differentially expressed proteins. **B** Heatmap of differentially expressed proteins. **C** BP analysis of differentially expressed proteins. **D** CC analysis of differentially expressed proteins. **E** MF analysis of differentially expressed proteins. **F** KEGG analysis of differentially expressed proteins

To understand the effects of DZP on the protein expression in the brain tissue of PD mice, we conducted a proteomics analysis. A total of 9393 proteins were detected. The results of the differential expression calculation showed that 207 differentially expressed proteins were detected in the DZP group compared to the PD group (Table 2), with 87 upregulated and 120 downregulated (Fig. 5A, B). GO analysis found that these differentially expressed proteins were mainly located in the cytoplasm and on the mitochondria and played a role in protein binding molecule functions to regulate apoptotic processes (Fig. 5C, E). KEGG analysis found that these proteins were involved in multiple pathways, including Pathways of neurodegeneration—multiple diseases, Huntington disease, Hippo signaling pathway, Parkinson’s disease, Alzheimer disease, ferroptosis, GABAergic synapse, amyotrophic lateral sclerosis (Fig. 5F).

**Table 2:** Differentially expressed proteins of DZP-treated PD mice in midbrain tissue

Accession	Gene symbol	Protein name	Significant
B2RXR4	2210408I21Rik	2210408I21Rik protein	Up
Q9 CQN3	Tomm6	Mitochondrial import receptor subunit TOM6 homolog	Up
Q8R2X8	Blzf1	Golgin-45	Up
Q3 TXJ4	Sar1a	GTP-binding protein SAR1a	Up
Q8 K3 J9	Gprc5c	G-protein coupled receptor family C group 5 member C	Up
Q9 WV27	Atp1a4	Sodium/potassium-transporting ATPase subunit alpha-4	Up
Q923 K4	Gtpbp3	tRNA modification GTPase GTPBP3, mitochondrial	Up
P97369	Ncf4	Neutrophil cytosol factor 4	Up
Q9 WUK6	Zbtb18	Zinc finger and BTB domain-containing protein 18	Up
Q9 JI10	Stk3	Serine/threonine-protein kinase 3	Up
P58158	B3 gat3	Galactosylgalactosylxylosylprotein 3-beta-glucuronosyltransferase 3	Up
B2RQG2	Phf3	PHD finger protein 3	Up
Q8BSE0	Rmdn2	Regulator of microtubule dynamics protein 2	Up
Q9 CPU2	Ndufb2	NADH dehydrogenase [ubiquinone] 1 beta subcomplex subunit 2, mitochondrial	Up
Q99LG1	Tmem51	Transmembrane protein 51	Up
A2 A472	Tox2	TOX high mobility group box family member 2	Up
O09005	Degs1	Sphingolipid delta(4)-desaturase DES1	Up
Q9 CZT5	Vasn	Vasorin	Up
Q571 F8	Gls2	Glutaminase liver isoform, mitochondrial	Up
Q5 FWH2	Unkl	Putative E3 ubiquitin-protein ligase UNKL	Up
Q8 K000	Sec16a	Sec16a protein (Fragment)	Up
Q11127	Fut4	Alpha-(1,3)-fucosyltransferase 4	Up
Q5U405	Tmprss13	Transmembrane protease serine 13	Up
Q8 CI15	Sphk1	Sphingosine kinase 1	Up
P59941	Sirt6	NAD-dependent protein deacylase sirtuin-6	Up
A0 A0R4 J0B1	Mark2	Non-specific serine/threonine protein kinase	Up
Q9D168	Ints12	Integrator complex subunit 12	Up
Q3 TN34	Micall2	MICAL-like protein 2	Up
E9PVU7	Pde4 d	Phosphodiesterase	Up
Q9 JKP5	Mbnl1	Muscleblind-like protein 1	Up
P56942	Pmch	Pro-MCH	Up
Q7 TST3	Samd10	Sterile alpha motif domain-containing protein 10	Up
A0 A0R4 J0E4	Ints7	Integrator complex subunit 7	Up
O35668	Hap1	Huntingtin-associated protein 1	Up
Q920Q6	Msi2	RNA-binding protein Musashi homolog 2	Up
Q80 WC7	Agfg2	Arf-GAP domain and FG repeat-containing protein 2	Up
Q9DAX9	Appbp2	Amyloid protein-binding protein 2	Up
S4R180	Snph	Syntaphilin (Fragment)	Up
Q3 TAS6	Emc10	ER membrane protein complex subunit 10	Up
Q9ESX0	Gphn	Gephyrin (Fragment)	Up
P97793	Alk	ALK tyrosine kinase receptor	Up
Q9 JIZ9	Plscr3	Phospholipid scramblase 3	Up
Q9QYN3	Klk11	Kallikrein-11	Up
Q8 K1X3	Tcf12	Tcf12 protein	Up
Q9R0Q4	Morf4 l2	Mortality factor 4-like protein 2	Up
Q9Z188	Dyrk1b	Dual specificity tyrosine-phosphorylation-regulated kinase 1B	Up
Q80ZD9	Amigo2	Amphoterin-induced protein 2	Up
Q8R3 V6	Cuedc1	CUE domain-containing protein 1	Up
Q9 CQA6	Chchd1	Small ribosomal subunit protein mS37	Up
O88819	Fut9	4-galactosyl-N-acetylglucosaminide 3-alpha-L-fucosyltransferase 9	Up
Q8BQP9	Rgs7bp	Regulator of G-protein signaling 7-binding protein	Up
Q5U5 V2	Hykk	Hydroxylysine kinase	Up
C9 K101	Mark1	Non-specific serine/threonine protein kinase	Up
Q3UQI9	Mindy4	Probable ubiquitin carboxyl-terminal hydrolase MINDY-4	Up
Q9ER97	Ngb	Neuroglobin	Up
G5E8B9	Zbtb11	Zinc finger and BTB domain-containing protein 11	Up
Q9 CXP8	Gng10	Guanine nucleotide-binding protein G(I)/G(S)/G(O) subunit gamma-10	Up
Q8 C4 V1	Arhgap24	Rho GTPase-activating protein 24	Up
Q8 K4G1	Ltbp4	Latent-transforming growth factor beta-binding protein 4	Up
P60904	Dnajc5	DnaJ homolog subfamily C member 5	Up
Q9D1P2	Kat8	Histone acetyltransferase KAT8	Up
Q3UQA7	Selenoh	Selenoprotein H	Up
P50114	S100b	Protein S100-B	Up
Q64191	Aga	N(4)-(beta-N-acetylglucosaminyl)-L-asparaginase	Up
A0 A991EKZ2	Fxr2	FMR1 autosomal homolog 2	Up
Q3 TH73	Ttyh2	Protein tweety homolog 2	Up
Q80 TA1	Selenoi	Ethanolaminephosphotransferase 1	Up
A2 CG63	Arid4b	AT-rich interactive domain-containing protein 4B	Up
Q3U4G3	Xxylt1	Xyloside xylosyltransferase 1	Up
O35492	Clk3	Dual specificity protein kinase CLK3	Up
Q8R3 N1	Nop14	Nucleolar protein 14	Up
Q99 JP4	Cdc26	Anaphase-promoting complex subunit CDC26	Up
Q6 A039	Tbc1 d12	TBC1 domain family member 12	Up
Q80 W04	Tmcc2	Transmembrane and coiled-coil domains protein 2	Up
Q7 TNP2	Ppp2r1b	Serine/threonine-protein phosphatase 2 A 65 kDa regulatory subunit A beta isoform	Up
Q6PGH2	Jpt2	Jupiter microtubule associated homolog 2	Up
G3UX33	Snx14	Sorting nexin 14	Up
A0 A571BEE9	4930517 N10Rik	RIKEN cDNA 4930517 N10 gene	Up
P26262	Klkb1	Plasma kallikrein	Up
Q5U4D8	Slc5a6	Sodium-dependent multivitamin transporter	Up
D3YZZ4	Spacdr	Sperm acrosome developmental regulator	Up
Q9 JHI2	Adat1	tRNA-specific adenosine deaminase 1	Up
A3 KFM7	Chd6	Chromodomain-helicase-DNA-binding protein 6	Up
A2 A5Y6	Mapt	Microtubule-associated protein	Up
Q6PAR0	Klhdc10	Kelch domain-containing protein 10	Up
P08122	Col4a2	Collagen alpha-2(IV) chain	Up
Q8BH49	Pheta1	Sesquipedalian-1	Up
Q9 JHH9	Copz2	Coatomer subunit zeta-2	Down
Q8 CIF6	Sidt2	SID1 transmembrane family member 2	Down
Q9Z126	Pf4	Platelet factor 4	Down
Q5EBQ0	Vdac3	Voltage-dependent anion channel 3	Down
Q9 CQB7	Lyrm1	LYR motif-containing protein 1	Down
P62315	Snrpd1	Small nuclear ribonucleoprotein Sm D1	Down
Q8BH88	Depdc1b	DEP domain-containing protein 1B	Down
Q91 VS7	Mgst1	Microsomal glutathione S-transferase 1	Down
Q8R4H9	Slc30a5	Proton-coupled zinc antiporter SLC30 A5	Down
O88572	Lrp6	Low-density lipoprotein receptor-related protein 6	Down
Q922Q9	Chid1	Chitinase domain-containing protein 1	Down
Q9 JJJ7	Porcn	Protein-serine O-palmitoleoyltransferase porcupine	Down
A8DUK2	Hbb-b1	Beta-globin	Down
Q91XI5	Dguok	Deoxyguanosine kinase 3	Down
Q80XI4	Pip4k2b	Phosphatidylinositol 5-phosphate 4-kinase type-2 beta	Down
Q9 CZR3	Tomm40 l	Mitochondrial import receptor subunit TOM40B	Down
Q7 TSH6	Scaf4	SR-related and CTD-associated factor 4	Down
Q9 CR67	Tmem33	Transmembrane protein 33	Down
Q91 WE6	Cdkal1	Threonylcarbamoyladenosine tRNA methylthiotransferase	Down
Q6GTY4	Sel1 l	Sel-1 suppressor of lin-12-like (C. elegans)	Down
P35802	Gpm6a	Neuronal membrane glycoprotein M6-a	Down
Q8 C460	Eri3	ERI1 exoribonuclease 3	Down
Q3 V0Y1	4930415L06Rik	Protein PPP4R3 C	Down
Q9Z0I6	Slfn2	Schlafen family member 2	Down
Q6P1Y8	Inpp4b	Type II inositol 3,4-bisphosphate 4-phosphatase	Down
Q4QRK2	Fech	Fech protein (fragment)	Down
A4 FU62	Iglv2	LOC207685 protein (fragment)	Down
P98192	Gnpat	Dihydroxyacetone phosphate acyltransferase	Down
E9Q2 W9	Actn4	Actinin alpha 4 (fragment)	Down
Q3 T1 F6	Mag	Myelin-associated glycoprotein	Down
Q9 CPU4	Mgst3	Glutathione S-transferase 3, mitochondrial	Down
P62141	Ppp1cb	Serine/threonine-protein phosphatase PP1-beta catalytic subunit	Down
Q3U962	Col5a2	Collagen alpha-2(V) chain	Down
O08738	Casp6	Caspase-6	Down
A0 A0B4 J1E2	Snw1	SNW domain-containing protein 1	Down
Q2PZL6	Fat4	Protocadherin Fat 4	Down
B7ZMV2	2810459M11Rik	Uncharacterized protein	Down
P01872	Ighm	Immunoglobulin heavy constant mu	Down
A0 A1B0GST8	Gm55978	Predicted gene, 55,978	Down
Q6DFW0	C9orf72	Guanine nucleotide exchange factor C9orf72 homolog	Down
Q3UEZ8	Slc10a4	Sodium/bile acid cotransporter 4	Down
Q91 V01	Lpcat3	Lysophospholipid acyltransferase 5	Down
Q8R2Q8	Bst2	Bone marrow stromal antigen 2	Down
Q60929	Mef2a	Myocyte-specific enhancer factor 2 A	Down
P53986	Slc16a1	Monocarboxylate transporter 1	Down
Q9D975	Srxn1	Sulfiredoxin-1	Down
Q62074	Prkci	Protein kinase C iota type	Down
P49769	Psen1	Presenilin-1	Down
Q8BSM7	Slc43a1	Large neutral amino acids transporter small subunit 3	Down
P00848	ATP6	ATP synthase subunit a	Down
A0 A1L1SUI3	Slc37a4	Solute carrier family 37 (glucose-6-phosphate transporter), member 4	Down
O70439	Stx7	Syntaxin-7	Down
Q8BGF9	Slc25a44	Solute carrier family 25 member 44	Down
P97441	Slc30a3	Probable proton-coupled zinc antiporter SLC30 A3	Down
Q8BGA5	Krr1	KRR1 small subunit processome component homolog	Down
P12710	Fabp1	Fatty acid-binding protein, liver	Down
B7ZNW4	Cox18	Cox18 protein	Down
Q78 JE5	Fbxo22	F-box only protein 22	Down
Q9DCC8	Tomm20	Mitochondrial import receptor subunit TOM20 homolog	Down
Q921Q3	Alg1	Chitobiosyldiphosphodolichol beta-mannosyltransferase	Down
O35344	Kpna3	Importin subunit alpha-4	Down
P58873	Rhbdl3	Rhomboid-related protein 3	Down
Q9 CPV9	P2ry12	P2Y purinoceptor 12	Down
Q61423	Kcna4	Potassium voltage-gated channel subfamily A member 4	Down
Q2 TA57	Asphd1	Aspartate beta-hydroxylase domain-containing protein 1	Down
A2 AEG3	Gpm6b	Glycoprotein m6b	Down
O55101	Syngr2	Synaptogyrin-2	Down
P62340	Tbpl1	TATA box-binding protein-like 1	Down
Q75 N73	Slc39a14	Metal cation symporter ZIP14	Down
Q80UK8	Ints2	Integrator complex subunit 2	Down
Q80 TL4	Phf24	PHD finger protein 24	Down
A1BN54	Actn1	Actinin, alpha 1	Down
A2 ANP1	Fam219a	2310028H24Rik protein	Down
Q8BR76	Tmem67	Meckelin	Down
A2 ALI5	Ajap1	Adherens junction-associated protein 1	Down
P35441	Thbs1	Thrombospondin-1	Down
A0 A6I8MWW1	Gas7	Growth arrest specific 7	Down
A2 AQ87	Shf	Src homology 2 domain containing F (Fragment)	Down
O70451	Slc16a7	Monocarboxylate transporter 2	Down
P01863	Ighg	Ig gamma-2 A chain C region, A allele	Down
Q6LD55	Apoa2	Apolipoprotein A-II	Down
Q9 CY97	Ssu72	RNA polymerase II subunit A C-terminal domain phosphatase SSU72	Down
A0 A0 F7R5U8	LC	MAb 110 light chain	Down
Q9EQ28	Pold3	DNA polymerase delta subunit 3	Down
P62137	Ppp1ca	Serine/threonine-protein phosphatase PP1-alpha catalytic subunit	Down
Q04692	Smarcad1	SWI/SNF-related matrix-associated actin-dependent regulator of chromatin subfamily A containing DEAD/H box 1	Down
Q66 T02	Plekhg5	Pleckstrin homology domain-containing family G member 5	Down
Q8 K4 T3	Stradb	STE20-related kinase adapter protein beta	Down
Q8BGS7	Cept1	Choline/ethanolaminephosphotransferase 1	Down
Q9D8 T7	Slirp	SRA stem-loop-interacting RNA-binding protein, mitochondrial	Down
A0 A571BEM7	Kcnab2	Voltage-gated potassium channel subunit beta-1	Down
Q9D9M2	Usp12	Ubiquitin carboxyl-terminal hydrolase 12	Down
Q9 JKV1	Adrm1	Proteasomal ubiquitin receptor ADRM1	Down
O89112	Lancl1	Glutathione S-transferase LANCL1	Down
Q8BP27	Sfr1	Swi5-dependent recombination DNA repair protein 1 homolog	Down
Q8R0X7	Sgpl1	Sphingosine-1-phosphate lyase 1	Down
Q8BIG7	Comtd1	Catechol O-methyltransferase domain-containing protein 1	Down
Q80 TH1	Dlg3	MKIAA1232 protein (fragment)	Down
P84086	Cplx2	Complexin-2	Down
P43006	Slc1a2	Excitatory amino acid transporter 2	Down
Q9 CXV1	Sdhd	Succinate dehydrogenase [ubiquinone] cytochrome b small subunit, mitochondrial	Down
Q9 CR25	Dph2	2-(3-amino-3-carboxypropyl)histidine synthase subunit 2	Down
B7ZC24	Ncoa5	Nuclear receptor coactivator 5	Down
Q9DB00	Gon4 l	GON-4-like protein	Down
Q80 T74	Klhl29	Kelch-like protein 29	Down
A2 AL36	Cntrl	Centriolin	Down
Q9 CZB0	Sdhc	Succinate dehydrogenase cytochrome b560 subunit, mitochondrial	Down
Q8 C726	Btbd9	BTB/POZ domain-containing protein 9	Down
Q9QUQ5	Trpc4	Short transient receptor potential channel 4	Down
Q99 KW5	Abhd4	Abhd4 protein (fragment)	Down
P14869	Rplp0	Large ribosomal subunit protein uL10	Down
P17047	Lamp2	Lysosome-associated membrane glycoprotein 2	Down
A0 A5 F8MPK1	Dpp10	Dipeptidylpeptidase 10	Down
Q925E1	Sntg1	Gamma-1-syntrophin	Down
B2RXE2	Slc9a5	Sodium/hydrogen exchanger 5	Down
Q9 CZT8	Rab3b	Ras-related protein Rab-3B	Down
Q3UMF9	Faxc	Failed axon connections homolog	Down
Q6GV12	Kdsr	3-ketodihydrosphingosine reductase	Down
A0 A087 WSP5	Stat1	Signal transducer and activator of transcription	Down
Q9R1X5	Abcc5	ATP-binding cassette sub-family C member 5	Down

### Analysis of active ingredients and target of DZP

**Fig. 6:**
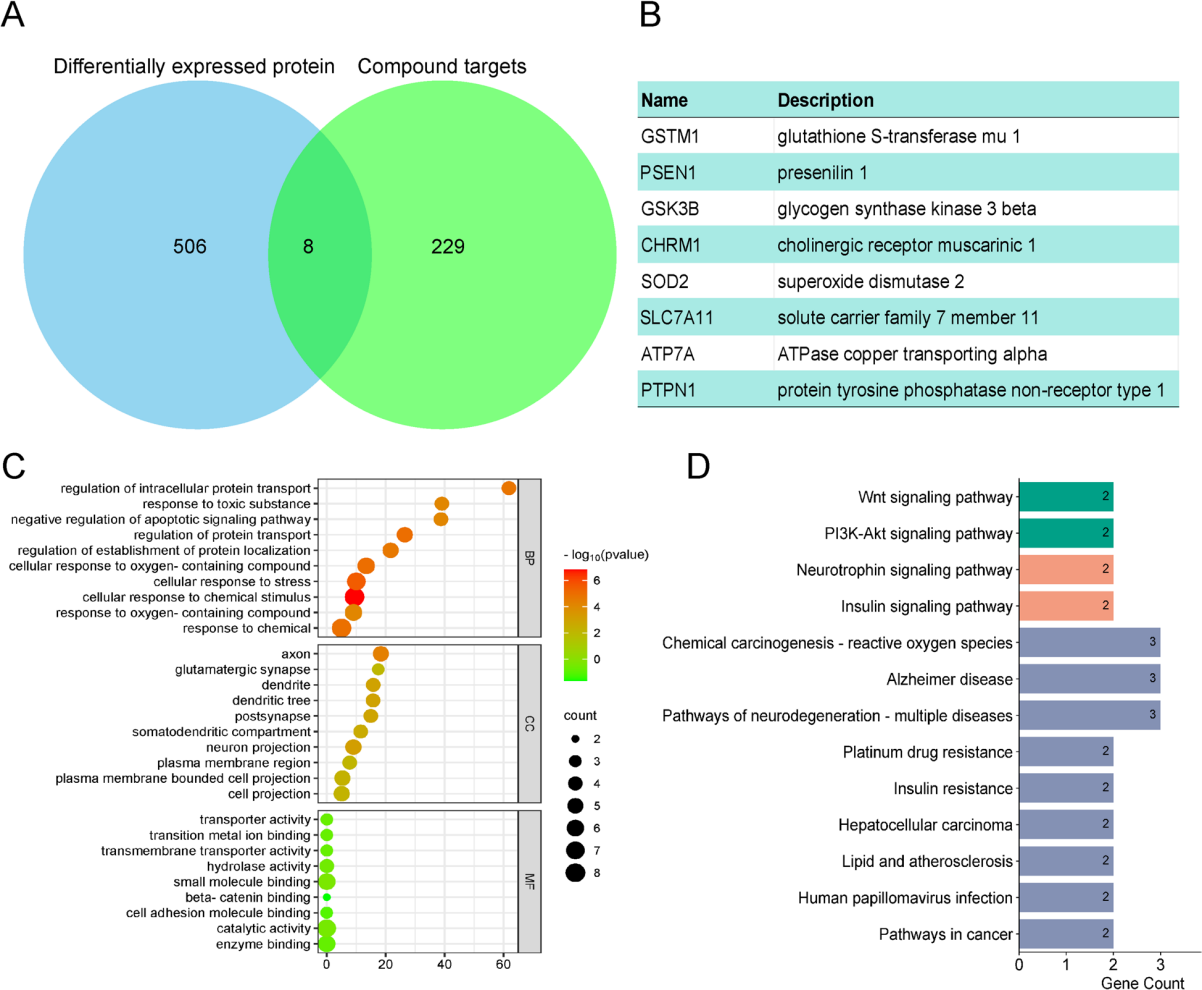
The multiple components and multiple target proteins Analysis of DZP for PD. **A** Intersection analysis of differentially expressed proteins in the DZP-treated PD mouse model and the targets affected by DZP in PD. **B** Detailed information on eight identified proteins. **C** GO analysis of the eight identified proteins. **D** KEGG pathway analysis of these eight proteins.

We conducted an analysis to identify the components and targets involved in DZP’s therapeutic effects on PD. Through proteomic profiling of DZP for PD model, as well as prediction of potential targets for DZP constituents, we found that eight molecules were consistently present across both datasets: ATP7 A, CHRM1, GSK3B, GSTM1, PSEN1, PTPN1, SLC7 A11 and SOD2 (Fig. 6A, B). Furthermore, GO and KEGG pathway analyses revealed their involvement in physiological processes such as reactive oxygen species regulation and neurodegenerative diseases (Fig. 6C, D).

**Fig. 7:**
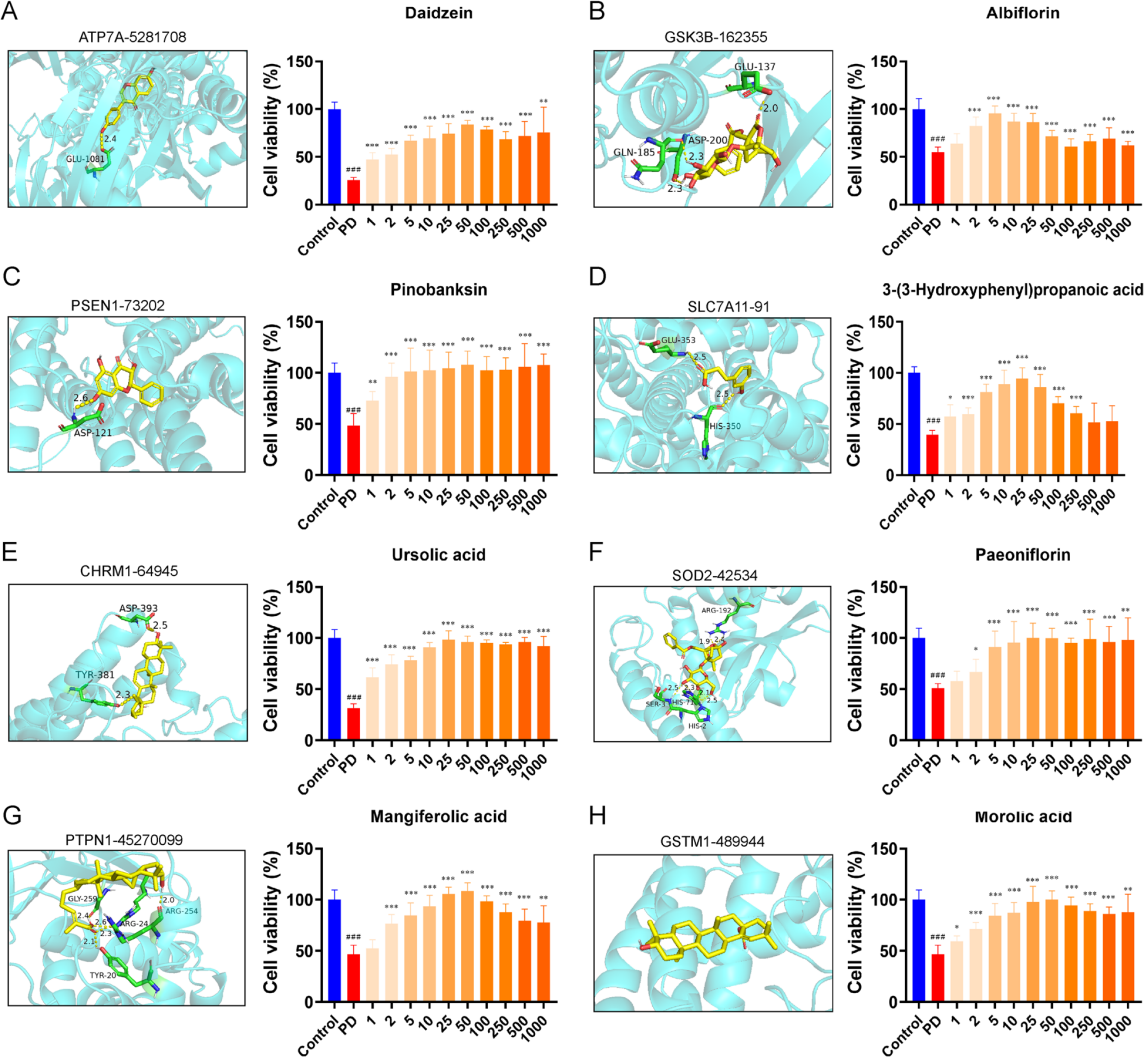
Molecular docking and neuroprotective activity verification of potential active ingredients of DZP. Molecular docking analysis and neuronal protective activity analysis were performed in vitro models of PD, including ATP7 A-Daidzein (**A**), GSK3B-Albiflorin (**B**), PSEN1-Pinobanksin (**C**), SLC7 A11–3-(3-Hydroxyphenyl) propanoic acid (**D**), CHRM1-Ursolic acid (**E**), SOD2-Paeoniflorin (**F**), PTPN1-Mangiferolic acid (**G**) and GSTM1-Morolic acid (**H**)

By examining compound-target interactions, we discovered specific compounds that interact with these molecules. Using molecular docking simulations to model compound-protein binding interactions and calculate binding affinities confirmed these interactions (Table 3): ATP7 A-5281708 [Daidzein, −7.27 ± 0.25 kcal/mol], CHRM1-64,945 [Ursolic acid, −10.08 ± 0.36 kcal/mol], GSK3B-162355 [Albiflorin, −8.18 ± 0.19 kcal/mol], GSTM1-489,944 [Morolic acid, −7.57 ± 0.23 kcal/mol], PSEN1-73,202 [Pinobanksin, −8.63 ± 0.29 kcal/mol], PTPN1-45270099[Mangiferolicacid, −8.78 ± 0.32 kcal/mol], SLC7 A11–91[3-(3-Hydroxyphenyl)-propionic acid, −5.59 ± 0.15 kcal/mol], SOD2–442534[Paeoniflorin, −6.47 ± 0.18 kcal/mol] (Fig. 7A–H). In an in vitro model of PD, MPP + significantly reduced the cellular activity of the neuronal cell SH-SY5Y. After small molecule therapy, neuronal activity was significantly increased in the treatment group and was associated with concentration dependence. In summary, the results suggest that substances of DZP such as Pinobanksin, Daidzein, Ursolic acid, and Albiflorinand others may modulate oxidative stress pathways through their interaction with PD target proteins.

**Table 3:** Multi-component and multi-target analysis of DZP for PD

Compounds	Formula	Protein target ID	Binding energy (kcal/mol)
Daidzein	C_15_H_10_O_4_	ATP7 A	−7.27 ± 0.25
Ursolic acid	C_30_H_48_O_3_	CHRM1	−10.08 ± 0.36
Albiflorin	C_23_H_28_O_11_	GSK3B	−8.18 ± 0.19
Morolic acid	C_30_H_48_O_3_	GSTM1	−7.57 ± 0.23
Pinobanksin	C_15_H_12_O_5_	PSEN1	−8.63 ± 0.29
Mangiferolic acid	C_30_H_48_O_3_	PTPN1	−8.78 ± 0.32
3-(3-Hydroxyphenyl) propanoic acid	C_9_H_10_O_3_	SLC7 A11	−5.59 ± 0.15
Paeoniflorin	C_23_H_28_O_11_	SOD2	−6.47 ± 0.18

### DZP inhibits neuronal ferroptosis by inhibiting cGAS-STING pathway

**Fig. 8:**
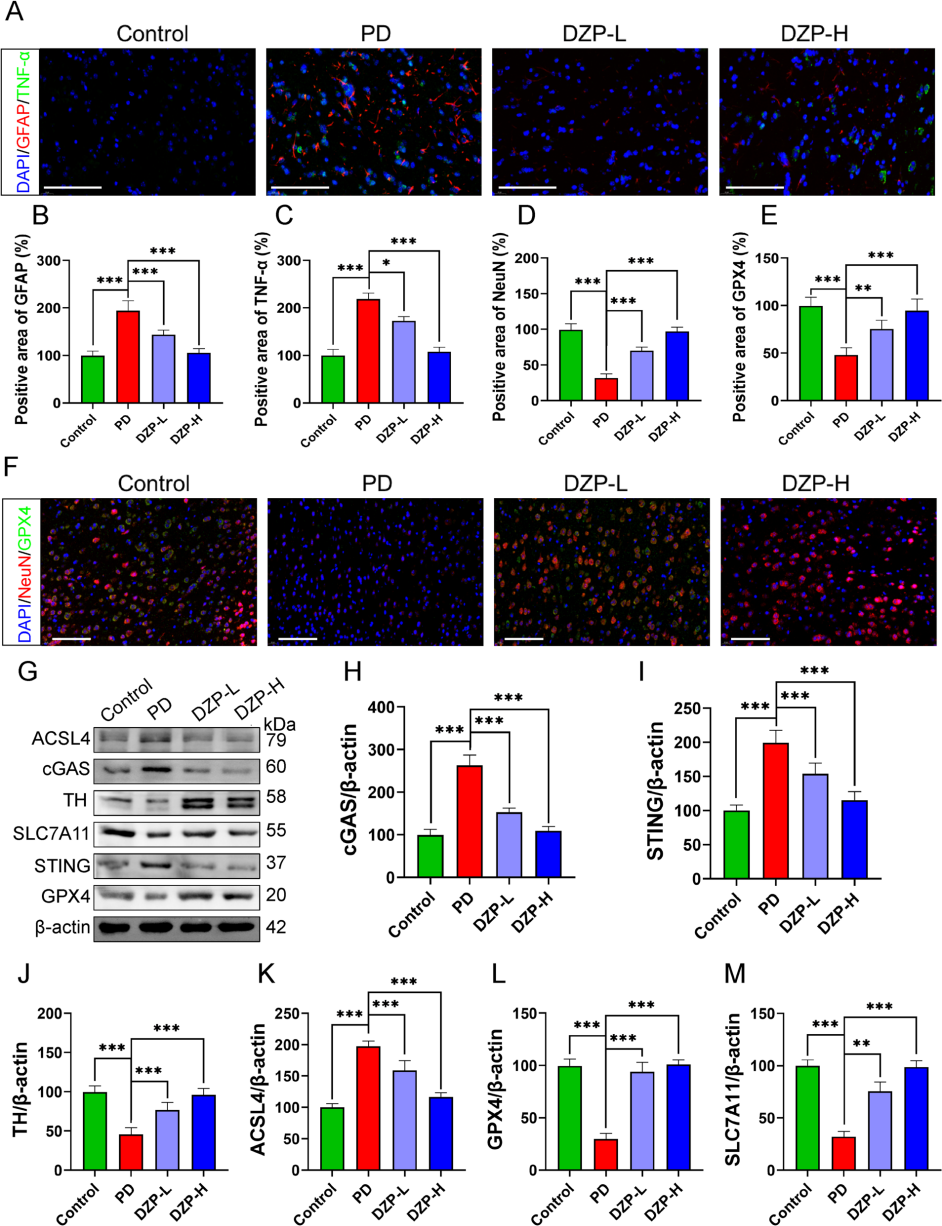
DZP inhibits the cGAS-STING pathway to suppress neuronal ferroptosis and neuroinflammation. **A** Immunofluorescence analysis of GFAP and TNF-α expression in the striatum of PD mice. Scale bars: 100 μm. **B** Statistical analysis of GFAP-positive regions. **C** Statistical analysis of TNF-α-positive regions. **D** Statistical analysis of NeuN-positive regions. **E** Statistical analysis of GPX4-positive regions. **F** Immunofluorescence analysis of NeuN and GPX4 expression in the striatum of PD mice. Scale bars: 100 μm. Western blot (WB) analysis of protein expression in the midbrain tissue of PD mice and its statistical analysis (**G**), including cGAS (**H**), STING (**I**), TH (**J**), ACSL4 (**K**), GPX4 (**L**) and SLC7A11 (**M**). The data are mean ± SD; *P < 0.05; **P < 0.01; ***P < 0.001 compared to the control group

The previous findings indicate that DZP may exert its therapeutic effects in PD through the pathways of oxidative stress, ferroptosis, and neuroinflammation. Astrocytes play a crucial role in regulating neuroinflammation. Our immunofluorescence results demonstrate that DZP significantly suppresses the expression of GFAP and TNF-α in the striatum region of PD mice, indicating its inhibitory effect on astrocyte proliferation and inflammation (Fig. 8A–C). The iron levels, MDA and ROS levels of midbrain tissue were significantly up-regulated in PD mice compared to controls, which were significantly down-regulated in the DZP-treated group (Fig. S2). Furthermore, our immunofluorescence results reveal a significant upregulation of NeuN and GPX4 expression following DZP treatment (Fig. 8D–F). GPX4 is a key regulator that mitigates ferroptosis. These results suggest that DZP attenuates neuronal ferroptosis to confer neuroprotective effects. Western blot analysis also shows restoration of TH levels in the DZP-treated group, indicative of neuronal recovery. Additionally, we observed significant upregulation of cGAS and STING in PD as well as downregulation after DZP treatment. Moreover, our findings demonstrate a significant increase in GPX4 and SLC7 A11 expression along with decreased ACSL4 expression following DZP treatment, confirming its substantial inhibition of ferroptosis (Fig. 8G–M). Collectively, these results support the notion that DZP inhibits neuronal ferroptosis and neuroinflammation by inhibiting the cGAS-STING pathway in PD mice.

## Discussion

Due to the limitations of existing clinical interventions, the development of novel drugs can help benefit patients with PD. TCM has a long history in the treatment of PD, and many herbal formulas have shown safety and efficacy, including Tianma Gouteng Decoction [14], Er Zhi Wan [15], Di Huang Yin [16], Buyang Huanwu Decoction [17], and Tongtian Oral Liquid [18].

Previous studies have indicated that these target proteins are involved in neuroinflammation or ferroptosis. ATP7 A is a copper (Cu)-ATPase involved in Cu export and plays a crucial role in maintaining cellular Cu homeostasis [19]. Abnormal Cu levels can affect iron metabolism in the brain, potentially triggering ferroptosis [20]. GSK3B, as an active mediator of neuroinflammation, has been widely discussed, with recent reports indicating its involvement in the activation of the NLRP3 inflammasome [21]. Additionally, GSK3B is also involved in the regulation of ferroptosis [22]. SLC7 A11, an essential component of the cystine/glutamate transporter (xCT), promotes glutathione synthesis by mediating cystine uptake and glutamate release and preventing lipid peroxidation-induced cell death [23]. Loss of SLC7 A11 is closely related to ferroptosis and has been observed in multiple PD studies [24–26]. SOD2, mainly located in the mitochondrial matrix, is crucial for cellular antioxidant defense, rescuing cells from peroxidation-induced ferroptosis [27, 28].

We found that DZP inhibited the cGAS-STING pathway. The cGAS-STING pathway driven immune responses play an important role in PD, and targeting cGAS-STING pathway serves as a promising strategy for PD treatment [29]. Its downstream reaction in the astrocyte YY1-LCN2 signaling cascade is involved in PD progression, whereas microglia STING activation contributes to neuroinflammation and neurodegeneration causing PD [30, 31]. Withaferin A, a natural product derived from the natural plant drunken eggplant, targets the regulation of DJ1-Nrf2-STING exerting neuroprotective effects in PD [32].

**Fig. 9:**
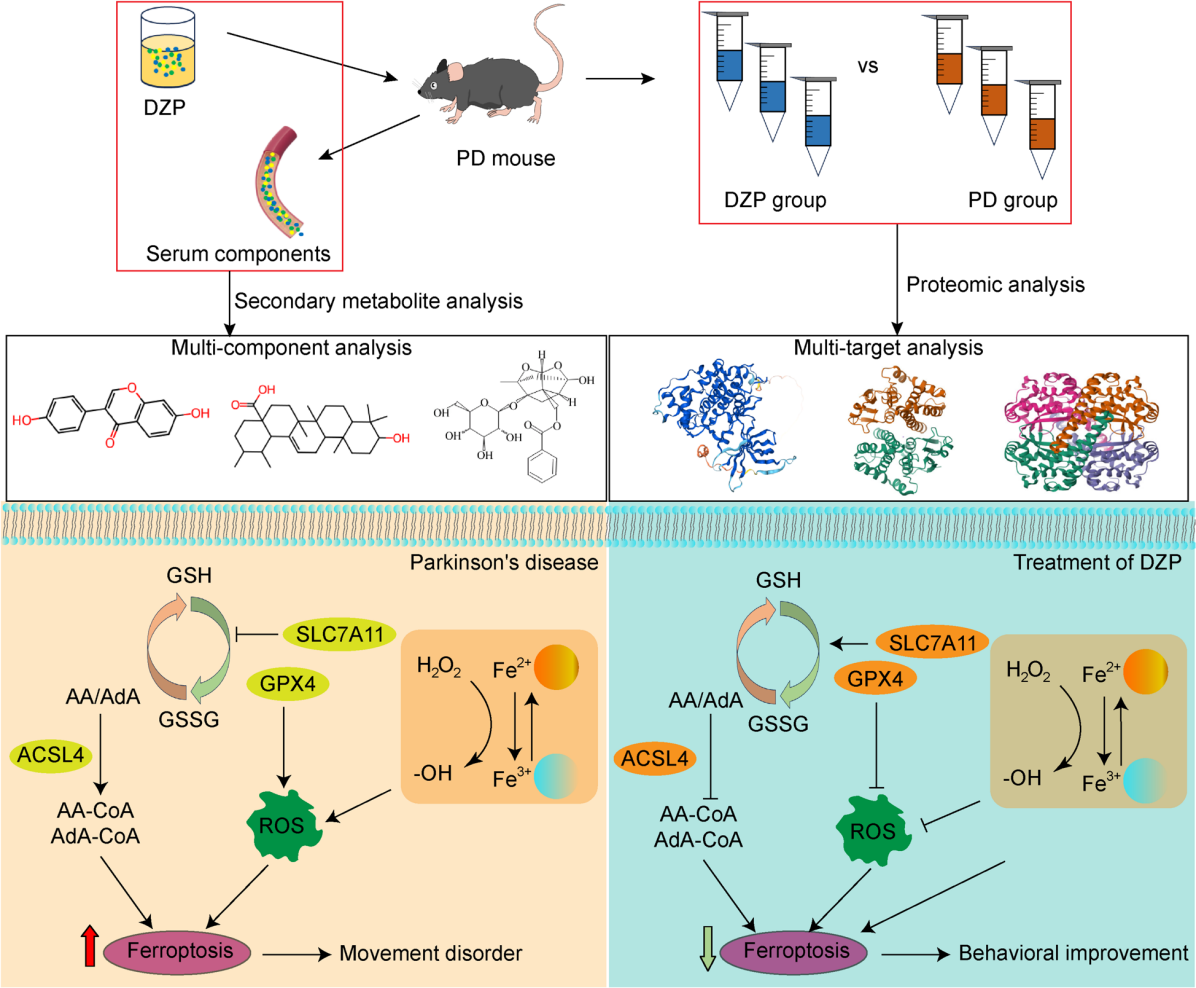
This figure highlights the mechanism by which TGD regulates ferroptosis in PD neurons.TGD directly binds to the pathological target proteins of PD through a variety of components and exerts neuroprotective effects by inhibiting ferroptosis, involving the inhibition of cGAS/STING pathway

Several compounds have been previously demonstrated to have therapeutic effects on PD [33]. Daidzein, a phytoestrogen, can improve motor dysfunction in MPTP-induced PD mice, reduce levels of pro-inflammatory mediators TNF-α, IL-1β, and IL-6, and restore dopamine levels [34]. In vitro studies have shown that Daidzein alleviates 6-OHDA-induced neurotoxicity in SH-SY5Y cells [35, 36]. Ursolic acid protects dopaminergic neurons and alleviates both motor and non-motor symptoms by reducing oxidative stress and neuroinflammation, enhancing autophagic clearance, and promoting mitochondrial biogenesis [37–39]. Totally-amounted glucosides of paeony (TGP), composed of albiflorin, paeoniflorin, oxypaeoniflorin, and benzoylpaeoniflorin, significantly improves motor coordination and striatal dopamine and its metabolite levels in PD mice [40]. Furthermore, Paeoniflorin reduces dopaminergic neuron degeneration by regulating neuroinflammation, apoptosis, and autophagy pathways [41–43]. 3-HPPA, a type of phenolic acid, can penetrate the blood–brain barrier. In vitro studies have shown that it effectively regulates α-syn misfolding, oligomerization, neurotoxicity, and brain-derived seeding activity [44]. Thus, the active ingredients of DZP, such as Daidzein, Ursolic acid, albiflorin, aeoniflorin, 3-HPPA, directly bind ATP7 A, GSK3B, SLC7 A11, SOD2, and inhibit neuronal ferroptosis in PD by significantly increasing the levels of GPX4 and SLC7 A11, reducing the levels of ACSL4 (Fig. 9).

## Conclusion

In this study, our findings demonstrated the neuroprotective effects of DZP in the MPTP-induced PD model. TGD directly binds to the pathological target proteins of PD through a variety of components and exerts neuroprotective effects by inhibiting ferroptosis, involving the inhibition of cGAS/STING pathway. We suggested that DZP is a potential therapy for PD which has an neuroprotective effect.

## Supplementary Information

Supplementary material 1.

Supplementary material 2.

## Data Availability

The datasets supporting this study’s conclusions are accessible through the corresponding author upon a reasonable request.

## Abbreviations

ANOVA: A one-way analysis of variance
DA: Dopaminergic
ECL: Enhanced chemiluminescence
GO: Gene ontology
IF: Immunofluorescence
LC–MS: Liquid chromatography–mass spectrometry
PBS: Phosphate buffer solution
PD: Parkinson’s disease
DZP: Dingzhen pill
IHC: Immunohistochemistry
PVDF: Polyvinylidene fluoride
KEGG: Kyoto Encyclopedia of Genes and Genomes
PPI: Protein–protein interaction
ROS: Reactive oxygen species
TCM: Traditional Chinese Medicine
TH: Tyrosine hydroxylase
MPTP: 1-Methyl-4-phenyl-1, 2, 3, 6-tetrahydro-pyridine

## References

[1] Global, regional, and national burden of neurological disorders, 1990–2016: a systematic analysis for the Global Burden of Disease Study 2016. Lancet Neurol, 2019, 18(5): 459–80. 10.1016/S1474-4422(18)30499-X PMC6459001 30879893

[2] Dorsey ER, Sherer T, Okun MS, et al. The emerging evidence of the Parkinson pandemic. J Parkinson’s Dis. 2018;8(s1):S3-s8. 30584159 10.3233/JPD-181474 PMC6311367

[3] Sveinbjornsdottir S. The clinical symptoms of Parkinson’s disease. J Neurochem. 2016;139(Suppl 1):318–24. 27401947 10.1111/jnc.13691

[4] Morris HR, Spillantini MG, Sue CM, et al. The pathogenesis of Parkinson’s disease. Lancet. 2024;403(10423):293–304. 38245249 10.1016/S0140-6736(23)01478-2

[5] Connolly BS, Lang AE. Pharmacological treatment of Parkinson disease: a review. JAMA. 2014;311(16):1670–83. 24756517 10.1001/jama.2014.3654

[6] Turgutalp B, Kizil C. Multi-target drugs for Alzheimer’s disease. Trends Pharmacol Sci. 2024;45(7):628–38. 38853102 10.1016/j.tips.2024.05.005

[7] Zhang Y, Xu X. Chinese herbal medicine in the treatment of depression in Parkinson’s disease: from molecules to systems. Front Pharmacol. 2022;13:879459. 35496318 10.3389/fphar.2022.879459 PMC9043316

[8] Yi P, Zhang Z, Huang S, et al. Integrated meta-analysis, network pharmacology, and molecular docking to investigate the efficacy and potential pharmacological mechanism of Kai-Xin-San on Alzheimer’s disease. Pharm Biol. 2020;58(1):932–43. 32956608 10.1080/13880209.2020.1817103 PMC7534219

[9] Jiang HP, Fang MQ. Dingzhen Decoction combined with levodopa/benserazide in Parkinson's disease: Efficacy and mechanism exploration in liver-kidney yin deficiency pattern. J Sichuan Tradit Chin Med. 2019; 37(10): 125-8.

[10] Zhang Y, Liang W, Luo X. Clinical controlled study on the treatment of Parkinson’s disease with Traditional Chinese Medicine Dingzhen Decoction. Liaoning J Tradit Chin Med. 2008;05:728–9.

[11] Kang H. The efficacy of Dingzhen pills combined with Pramipexole in the treatment of Parkinson’s disease patients. Med J Chin People’s Health. 2022;34(14):103–6.

[12] Huo Q, Tan F, Lin D, et al. The effects of Dingzhen pills on neurotransmitters and oxidative stress in dopaminergic neurons of substantia nigra in a rat model of Parkinson’s disease. Chin Tradit Pat Med. 2020;42(03):604–10.

[13] Cao J, Li C, Cui Z, et al. Spatial transcriptomics: a powerful tool in disease understanding and drug discovery. Theranostics. 2024;14(7):2946–68. 38773973 10.7150/thno.95908 PMC11103497

[14] Lei T, Fu GS, Xue X, et al. Tianma Gouteng Decoction improve neuronal synaptic plasticity and oligodendrocyte apoptosis in Parkinson’s disease mice. Phytomedicine. 2025. 10.1016/j.phymed.2025.156553. 40023970 10.1016/j.phymed.2025.156553

[15] Pan B, Niu B, He Y, et al. Integrative multilevel exploration of the mechanism by which Er-Zhi-Wan alleviates the Parkinson’s disease (PD)-like phenotype in the MPTP-induced PD mouse model. Biomed Pharmacother. 2023;165:115021. 37348406 10.1016/j.biopha.2023.115021

[16] Wu Y, Liu H, Wang Y, et al. DiHuangYin decoction protects dopaminergic neurons in a Parkinson’s disease model by alleviating peripheral inflammation. Phytomedicine. 2022;105:154357. 35933898 10.1016/j.phymed.2022.154357

[17] Hu J, Li P, Zhao H, et al. Alterations of gut microbiota and its correlation with the liver metabolome in the process of ameliorating Parkinson’s disease with Buyang Huanwu decoction. J Ethnopharmacol. 2024;318(Pt A):116893. 37423520 10.1016/j.jep.2023.116893

[18] Dongjie S, Rajendran RS, Xia Q, et al. Neuroprotective effects of Tongtian oral liquid, a Traditional Chinese Medicine in the Parkinson’s disease-induced zebrafish model. Biomed Pharmacother. 2022;148:112706. 35152046 10.1016/j.biopha.2022.112706

[19] Locatelli M, Farina C. Role of copper in central nervous system physiology and pathology. Neural Regen Res. 2025;20(4):1058–68. 38989937 10.4103/NRR.NRR-D-24-00110 PMC11438321

[20] SKJøRRINGE T, MøLLER LB, MOOS T. Impairment of interrelated iron-and copper homeostatic mechanisms in brain contributes to the pathogenesis of neurodegenerative disorders. Front Pharmacol. 2012;3:169. 23055972 10.3389/fphar.2012.00169 PMC3456798

[21] KHAN SS, JANRAO S, SRIVASTAVA S, et al. GSK-3β: an exuberating neuroinflammatory mediator in Parkinson’s disease. Biochem Pharmacol. 2023;210:115496. 36907495 10.1016/j.bcp.2023.115496

[22] Gui J, Wang L, Liu J, et al. Ambient particulate matter exposure induces ferroptosis in hippocampal cells through the GSK3B/Nrf2/GPX4 pathway. Free Radical Biol Med. 2024;213:359–70. 38290604 10.1016/j.freeradbiomed.2024.01.045

[23] Wang ZL, Yuan L, Li W, et al. Ferroptosis in Parkinson’s disease: glia-neuron crosstalk. Trends Mol Med. 2022;28(4):258–69. 35260343 10.1016/j.molmed.2022.02.003

[24] Zhang X, Li G, Chen H, et al. Targeting NKAα1 to treat Parkinson’s disease through inhibition of mitophagy-dependent ferroptosis. Free Radical Biol Med. 2024;218:190–204. 38574977 10.1016/j.freeradbiomed.2024.04.002

[25] Li M, Zhang J, Jiang L, et al. Neuroprotective effects of morroniside from Cornus officinalis sieb. Et zucc against Parkinson’s disease via inhibiting oxidative stress and ferroptosis. BMC Complement Med Ther. 2023;23(1):218. 37393274 10.1186/s12906-023-03967-0 PMC10314491

[26] Yao Z, Jia F, Wang S, et al. The involvement of IRP2-induced ferroptosis through the p53-SLC7A11-ALOX12 pathway in Parkinson’s disease. Free Radical Biol Med. 2024;222:386–96. 38936518 10.1016/j.freeradbiomed.2024.06.020

[27] Hu D, Sun X, Liao X, et al. Alpha-synuclein suppresses mitochondrial protease ClpP to trigger mitochondrial oxidative damage and neurotoxicity. Acta Neuropathol. 2019;137(6):939–60. 30877431 10.1007/s00401-019-01993-2 PMC6531426

[28] Ruszkiewicz J, Albrecht J. Changes in the mitochondrial antioxidant systems in neurodegenerative diseases and acute brain disorders. Neurochem Int. 2015;88:66–72. 25576182 10.1016/j.neuint.2014.12.012

[29] Huang YG, Liu BY, Sinha SC, et al. Mechanism and therapeutic potential of targeting cGAS-STING signaling in neurological disorders. Mol Neurodegener. 2023. 10.1186/s13024-023-00672-x. 37941028 10.1186/s13024-023-00672-x PMC10634099

[30] Jiang SY, Tian T, Yao H, et al. The cGAS-STING-YY1 axis accelerates progression of neurodegeneration in a mouse model of Parkinson’s disease via LCN2-dependent astrocyte senescence. Cell Death Differ. 2023;30(10):2280–92. 37633968 10.1038/s41418-023-01216-y PMC10589362

[31] Ma CM, Liu Y, Li S, et al. Microglial cGAS drives neuroinflammation in the MPTP mouse models of Parkinson’s disease. Cns Neurosci Ther. 2023;29(7):2018–35. 36914567 10.1111/cns.14157 PMC10324349

[32] Zhao M, Wang BW, Zhang CY, et al. The DJ1-Nrf2-STING axis mediates the neuroprotective effects of Withaferin A in Parkinson’s disease. Cell Death Differ. 2021;28(8):2517–35. 33762743 10.1038/s41418-021-00767-2 PMC8329302

[33] Lei T, Li CF, Liu Y, et al. Microfluidics-enabled mesenchymal stem cell derived neuron like cell membrane coated nanoparticles inhibit inflammation and apoptosis for Parkinson’s Disease. J Nanobiotechnol. 2024. 10.1186/s12951-024-02587-1. 10.1186/s12951-024-02587-1 PMC11197265 38918856

[34] Wu Q, Wang M, Chen W, et al. Daidzein exerts neuroprotective activity against MPTP-induced Parkinson’s disease in experimental mice and lipopolysaccharide-induced BV2 microglial cells. J Biochem Mol Toxicol. 2022;36(2):e22949. 34850494 10.1002/jbt.22949

[35] Ko YH, Kwon SH, Kim SK, et al. Protective effects of 6,7,4’-trihydroxyisoflavone, a major metabolite of daidzein, on 6-hydroxydopamine-induced neuronal cell death in SH-SY5Y human neuroblastoma cells. Arch Pharmacal Res. 2019;42(12):1081–91. 10.1007/s12272-019-01191-4 31705299

[36] Ko YH, Kim SK, Kwon SH, et al. 7,8,4’-Trihydroxyisoflavone, a metabolized product of daidzein, attenuates 6-hydroxydopamine-induced neurotoxicity in SH-SY5Y Cells. Biomol Ther. 2019;27(4):363–72. 10.4062/biomolther.2018.211 PMC6609108 30866601

[37] Zahra W, Rai SN, Birla H, et al. Neuroprotection of rotenone-induced Parkinsonism by Ursolic acid in PD mouse model. CNS Neurol Disord. 2020;19(7):527–40. 10.2174/1871527319666200812224457 32787765

[38] Bang Y, Kwon Y, Kim M, et al. Ursolic acid enhances autophagic clearance and ameliorates motor and non-motor symptoms in Parkinson’s disease mice model. Acta Pharmacol Sin. 2023;44(4):752–65. 36138143 10.1038/s41401-022-00988-2 PMC10042858

[39] Peshattiwar V, Muke S, Kaikini A, et al. Mechanistic evaluation of Ursolic acid against rotenone induced Parkinson’s disease—emphasizing the role of mitochondrial biogenesis. Brain Res Bull. 2020;160:150–61. 32147532 10.1016/j.brainresbull.2020.03.003

[40] Zheng M, Liu C, Fan Y, et al. Total glucosides of paeony (TGP) extracted from Radix Paeoniae Alba exerts neuroprotective effects in MPTP-induced experimental parkinsonism by regulating the cAMP/PKA/CREB signaling pathway. J Ethnopharmacol. 2019;245:112182. 31445131 10.1016/j.jep.2019.112182

[41] Liu HQ, Zhang WY, Luo XT, et al. Paeoniflorin attenuates neuroinflammation and dopaminergic neurodegeneration in the MPTP model of Parkinson’s disease by activation of adenosine A1 receptor. Br J Pharmacol. 2006;148(3):314–25. 16582933 10.1038/sj.bjp.0706732 PMC1751566

[42] Zheng M, Liu C, Fan Y, et al. Neuroprotection by Paeoniflorin in the MPTP mouse model of Parkinson’s disease. Neuropharmacology. 2017;116:412–20. 28093210 10.1016/j.neuropharm.2017.01.009

[43] Cao BY, Yang YP, Luo WF, et al. Paeoniflorin, a potent natural compound, protects PC12 cells from MPP+ and acidic damage via autophagic pathway. J Ethnopharmacol. 2010;131(1):122–9. 20558269 10.1016/j.jep.2010.06.009

[44] Yamasaki TR, Ono K, Ho L, et al. Gut microbiome-modified polyphenolic compounds inhibit α-synuclein seeding and spreading in α-synucleinopathies. Front Neurosci. 2020;14:398. 32431588 10.3389/fnins.2020.00398 PMC7212829

