# Supplementary material for: Dingzhen pills inhibit neuronal ferroptosis and neuroinflammation by inhibiting the cGAS-STING pathway for Parkinson’s disease mice
Source: Chin Med. 2025 Jun 16;20:87. doi: 10.1186/s13020-025-01135-9 (PMC12168260; doi:10.1186/s13020-025-01135-9)
Supplement: Supplementary file 1 — Supplementary material 1. [file 13020_2025_1135_MOESM1_ESM.docx]

**Dingzhen pills inhibit neuronal ferroptosis and neuroinflammation by inhibiting the cGAS-STING pathway for Parkinson's disease mice**

**Gaoshuang Fu^1^, Ting Li^2^, Yukun Zhao^1^, Shuai Zhang^1^, Xin Xue^1^, Yuqin Yang^1^, Tingyu Li^1^, Shaohan Luo^1^, Guangxin Yue^1*^, Tong Lei^1*^**

^1^Institute of Basic Theory for Chinese Medicine, China Academy of Chinese Medical Sciences, Beijing, 100700, China.

^2^Dongzhimen Hospital, Beijing University of Chinese Medicine, Beijing, 100700, China.

**Supplementary document materials**


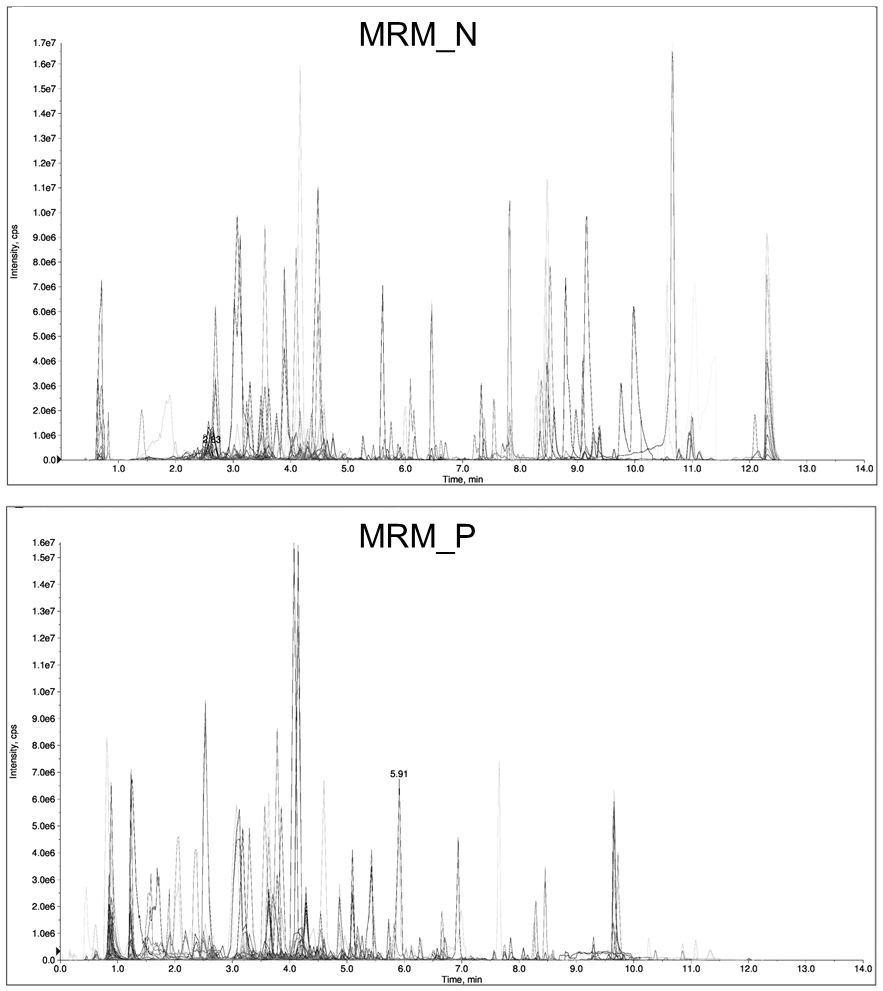


**Fig S1. Total ion chromatograms of mixed samples in negative (MRM_N) and positive (MRM_P) ion mode in UPLC-Q-Orbitrap HRMS.** The mixed samples included DZP decoction, mouse serum samples from PD mice in the DZP treatment group, and mouse serum samples from the PD group.


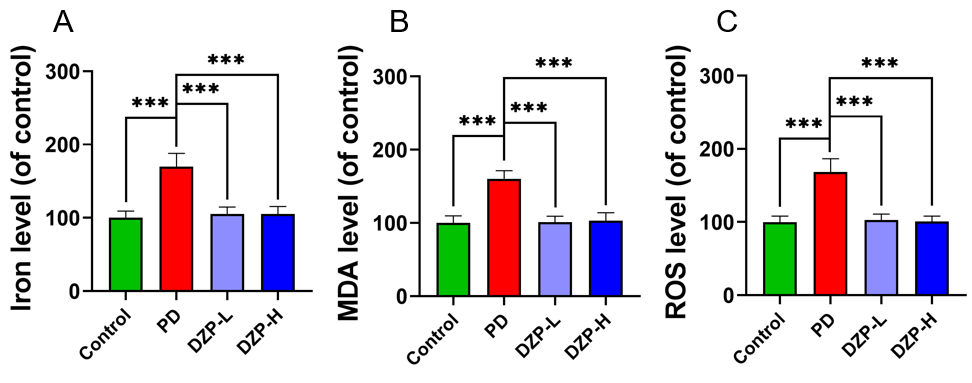


**Fig S2. The ferroptosis-related characterize of mouse midbrain tissue.** Iron ion (A), MDA (B), and ROA (C) levels in midbrain tissues from all groups.
